# Excess water availability in northern mid-high latitudes contiguously migrated from ocean under climate change

**DOI:** 10.1126/sciadv.adv0282

**Published:** 2025-08-13

**Authors:** Yansong Guan, Xihui Gu, Lunche Wang, Tianjun Zhou, Jun Xia, Dabang Jiang, Louise J. Slater, Luis Gimeno, Yadu Pokhrel, Gabriele Villarini, Jong-Seong Kug, Seok-Woo Son, Richard P. Allan, Jianfeng Li, Thian Yew Gan, Yinxue Liu, Dongdong Kong, Xiang Zhang, Xiangsen Cui

**Affiliations:** ^1^ State Key Laboratory of Geomicrobiology and Environmental Changes, China University of Geosciences, Wuhan, China.; ^2^ Hubei Key Laboratory of Regional Ecology and Environmental Change, School of Geography and Information Engineering, China University of Geosciences, Wuhan 430074, China.; ^3^ Institute of Atmospheric Physics, Chinese Academy of Sciences, Beijing, China.; ^4^ University of the Chinese Academy of Sciences, Beijing, China.; ^5^ State Key Laboratory of Water Resources and Hydropower Engineering Science, Wuhan University, Wuhan, China.; ^6^ School of Geography and the Environment, University of Oxford, Oxford, UK.; ^7^ Centro de Investigaciόn Mariña, Environmental Physics Laboratory, Universidade de Vigo, Ourense, Spain.; ^8^ Galician Supercomputing Center (CESGA), Santiago de Compostela, Spain.; ^9^ Department of Civil and Environmental Engineering, Michigan State University, East Lansing, MI, USA.; ^10^ Department of Civil and Environmental Engineering, Princeton University, Princeton, NJ, USA.; ^11^ High Meadows Environmental Institute, Princeton University, Princeton, NJ, USA.; ^12^ School of Earth and Environmental Sciences, Seoul National University, Seoul, South Korea.; ^13^ Department of Meteorology and National Centre for Earth Observation, University of Reading, Reading, UK.; ^14^ Department of Geography and Resource Management, The Chinese University of Hong Kong, Shatin, Hong Kong SAR, China.; ^15^ Department of Civil and Environmental Engineering, University of Alberta, Edmonton, AB, Canada.; ^16^ Geography and Environment, Loughborough University, Loughborough, UK.; ^17^ Department of Atmospheric Science, School of Environmental Studies, China University of Geosciences, Wuhan 430074, China.; ^18^ National Engineering Research Center of Geographic Information System, School of Geography and Information Engineering, China University of Geosciences, Wuhan 430074, China.

## Abstract

Terrestrial water availability sustains livelihoods, socioeconomic development, and ecosystems. Despite an understanding of contributions of oceanic moisture to terrestrial hydroclimatic extremes, whether surpluses of terrestrial water availability migrate directly and contiguously from the ocean and the influence of climate change on this process remain unclear. Here, we use a coherent feature-tracking method to identify ocean-to-land water availability surpluses (OWASs), characterized by spatiotemporally contiguous migration of excess atmospheric freshwater (precipitation-minus-evapotranspiration) from ocean to land. Over the past several decades, especially in northern mid-high latitudes (NMHL; above 48°N), OWASs have exhibited longer persistence, wider areal extent, and greater intensity than those developed solely over land. These landward migrations are associated with seasonal Atlantic teleconnection and Pacific circulation shift. Under the business-as-usual scenario, these two processes are projected to be enhanced, markedly increasing OWAS characteristics in NMHL driven by thermodynamic atmospheric responses to future warming. Intensified OWASs may not only help alleviate long-term droughts but also have the potential to accentuate pluvial risks.

## Teaser

Climate change can cause excess freshwater in the northern mid-high latitudes to continuously migrate from the ocean.

## INTRODUCTION

Terrestrial water availability is the net difference between freshwater supply from precipitation and demand from evapotranspiration [i.e., precipitation-minus-evapotranspiration ( PME)], representing the overall state of hydrological cycle (*1*, *2*). As a critical resource supporting agriculture, industry, and ecosystems, the dynamics of water availability are especially crucial in densely populated and water-scarce regions (*3*, *4*). On land, water availability corresponds to both runoff ( R ) and fluctuations in terrestrial water storage ( TWS ), as described by the water balance equation ( PME=R+∆TWS ) (*2*). Over the ocean, it represents atmospheric freshwater, approximately equaling changes in vertically integrated vapor moisture ( Q ) based on the atmospheric moisture budget ( PME≈−∇·Q ) (*5*, *6*). Negative water availability anomalies (WAAs) indicate terrestrial water depletion or oceanic moisture deficits and vice versa. The oceans, as the central hub of the global hydrological cycle, are the primary moisture source for the Earth’s land surface (*7*, *8*). Numerous studies have pointed to anomalous changes in terrestrial water availability that can often be traced back to contributions from the oceans along the moisture pathways (*9*, *10*). However, our understanding of the seasonal contiguous migration of water availability extremes from ocean to land remains limited. Recent research indicates that seasonal large-scale deficits in water availability over the North Atlantic can persist for several months and directly migrate to the Tibetan Plateau, modulating regional variations in TWS (*11*). Although the contribution of oceanic moisture to terrestrial hydroclimatic extremes is essential, we have not yet carefully explored terrestrial water availability surpluses directly migrated from the oceans. Such ocean-to-land transboundary migrations have the potential not only to alleviate long-term droughts but also to trigger pluvials (extreme wet conditions, that is, the opposite of droughts), posing challenges for regional water resource regulation, reservoir operations, and disaster risk management.

Anthropogenic warming substantially enhances global atmospheric moisture content and transport, amplifying the intensity and variability of the terrestrial hydrological cycle (*12*–*14*). These changes result not only in long-term changes in water availability but also in pronounced alterations to its seasonality (*15*, *16*). Enhanced seasonality implies that water availability surpluses become increasingly frequent during wet periods, leading to abrupt increases in runoff, heightened pluvial risks, and greater complexity in infrastructure management and disaster preparedness (*17*, *18*). Warming-induced amplification of the land-sea thermal contrast is driving substantial transport of oceanic moisture toward the land (*19*, *20*), particularly in northern mid-high latitudes (NMHL) (*21*). Previous studies have shown that warming-induced increases in moisture from the northern Atlantic are likely to contribute to rising freshwater over Europe in a warmer future (*22*–*24*). Similarly, anthropogenically intensified moisture transport from the Pacific is projected to increase water availability in western North America (*25*–*27*). Given the frequent and exacerbated terrestrial water availability extremes, it is more likely that in a warmer climate, not only deficits but also frequent surpluses in terrestrial water availability may be projected to directly migrate from the oceans. However, it is still unclear how anthropogenic forcings (ANT) influence the transboundary migration of water availability surpluses from ocean to land and the mechanisms driving this process.

**Fig. 1.**
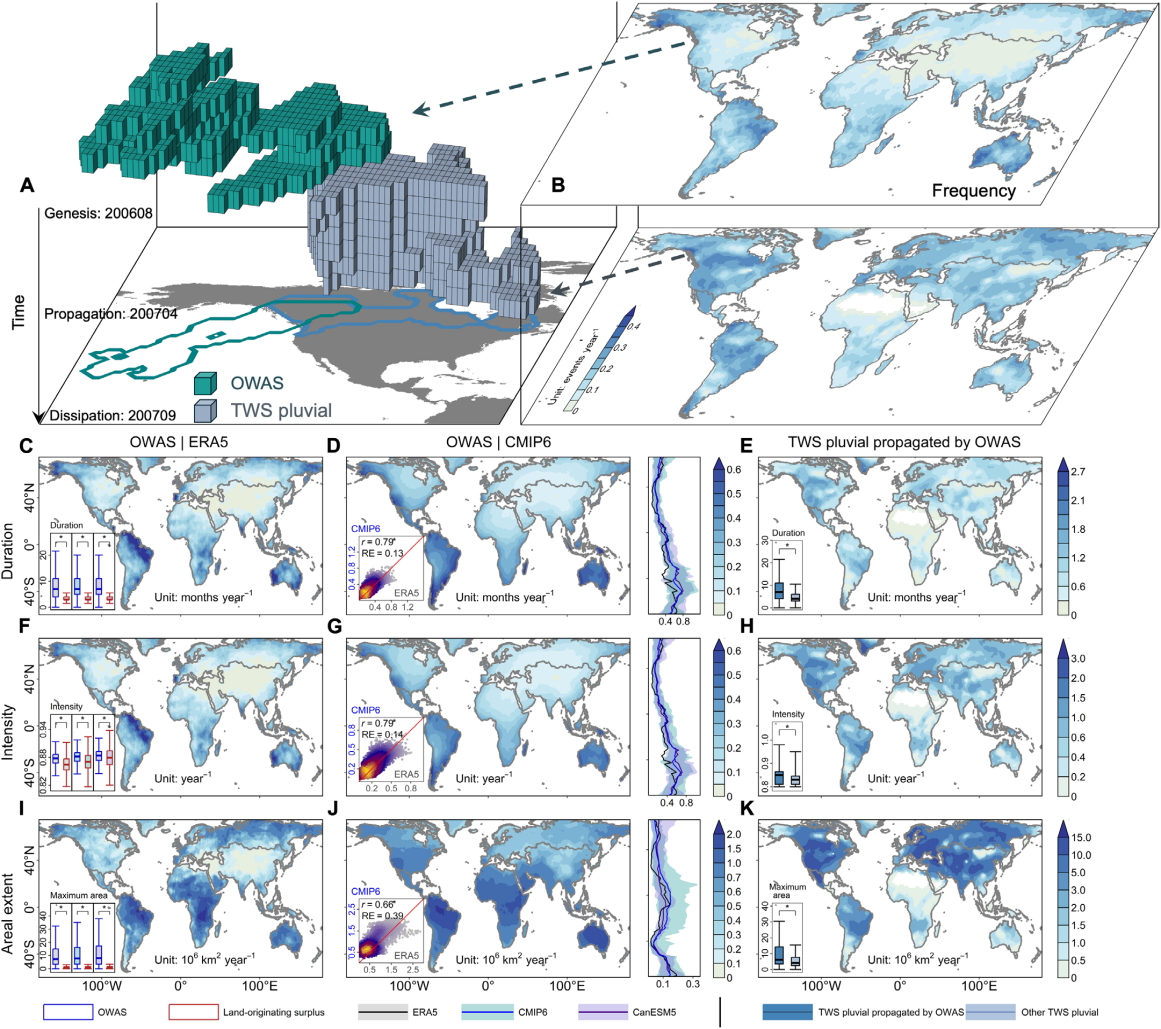
Climatological characteristics of OWAS during 1961–2020. (**A**) Three-dimensional evolution of OWAS and OWAS-propagated spatiotemporally contiguous TWS pluvial during August 2006–September 2007. (**B**) Maps show the frequency of OWASs (top) from ERA5 and OWAS-propagated TWS pluvial events (bottom) from GTWS-MLrec globally during 1961–2020. (**C** to **K**) Same as (B), but for duration [(C) to (E)], intensity [(F) to (H)], and areal extent [(I) to (K)] of OWASs from ERA5 and the CMIP6 multimodel mean, as well as those of OWAS-propagated TWS pluvial events from GTWS-MLrec. The bottom-left inset boxplots in [(C), (F), and (I)] represent the characteristics of OWASs and land-originating water availability surpluses from ERA5, 32 CMIP6 models, and 50 CanESM5 ensemble members. The inset scatterplots in [(D), (G), and (J)] represent the OWAS characteristics in each grid cell from CMIP6 multimodel mean and ERA5. The symbol “RE” indicates the relative error. The lines accompanying the maps in [(D), (G), and (J)] show the zonal means of the climatological OWAS characteristics from ERA5, CMIP6, and CanESM5. The blue (purple) shading shows the spread of zonal mean values for 90% of the CMIP6 models (CanESM5 ensemble members). The inset boxplots in [(E), (H), and (K)] represent the characteristics of OWAS-propagated TWS pluvial events and other TWS pluvial events. Symbol “*” indicates a significant difference between distributions (Kolmogorov-Smirnov test, *P* < 0.05) or a significant correlation (Pearson’s correlation, *P* < 0.001). The top to bottom boxplot bounds represent the value of Q3 + 1.5 × IQR, third quartile (Q3), median (horizontal line), first quartile (Q1), and Q1 − 1.5 × IQR, respectively, where IQR denotes the interquartile range (Q3 − Q1).

Here, we focus on the spatiotemporally contiguous seasonal migration of ocean-to-land water availability surpluses (OWASs; see Fig. 1A for an example). By quantifying changes in the frequency, duration, intensity, and areal extent of OWASs from reanalysis datasets (1961–2020) and climate model simulations (1921–2100), we investigate the anthropogenic influence on these OWAS characteristics across the different Giorgi climate regions from the Intergovernmental Panel on Climate Change (*28*) based on the sixth Coupled Model Intercomparison Project (CMIP6; table S1) (*29*). We further identify two primary landward migration routes of OWASs in NMHL and their associated atmospheric mechanisms. Last, we project future relative changes in these OWAS characteristics under an extreme emissions scenario [i.e., business-as-usual scenario, Shared Socioeconomic Pathway 5 (SSP5) and Representative Concentration Pathway 8.5 (RCP8.5) (SSP585)] and then quantify contributions of atmospheric thermodynamic and dynamic drivers.

## RESULTS

### Responses of historical OWASs to anthropogenic forcings

In this study, the detection of spatiotemporally contiguous water availability surpluses involves two main steps: identifying local surpluses based on a standardized water availability index (SWAI > 0.8) at each grid cell and then clustering them by a coherent feature-tracking method (Materials and Methods). On the basis of the state-of-the-art reanalysis dataset [fifth generation of the European Centre for Medium Range Weather Forecasts Reanalysis (ERA5); Materials and Methods], we identified 1053 terrestrial water availability surplus events from 1961 to 2020. These events were categorized into two types based on their origins, i.e., OWASs and land-originating surplus events. Over the six decades, global OWASs (582 events) are significantly more persistent (+133.3%) and extensive (+649.0%) than land-originating events (471 events) (Fig. 1, C, F, and I). OWASs originating from and developing across the Atlantic and the northern Pacific tend to occur more frequently, persistently, intensely, and extensively in NMHL (above 48°N), South America, and Australia compared to other regions (Fig. 1B and fig. S1). We also used the Japanese 55-year Reanalysis (JRA55) and Modern-Era Retrospective analysis for Research and Applications, version 2 (MERRA2) reanalysis datasets (Materials and Methods) to confirm ERA5-based results and found high spatial pattern correlations (*r* > 0.79; *P* < 0.001) of OWAS frequency among these datasets.

Terrestrial water availability surpluses during wet periods may cause abrupt increases in runoff and heighten fluvial risks (*17*, *18*). In addition, these surpluses can contribute to the generation of TWS pluvials (*30*). Using the same approach as OWASs, we identified spatiotemporally contiguous TWS pluvial events and tracked the propagation from OWASs to TWS pluvial events according to certain spatiotemporal overlap rules (*31*, *32*). For instance, the terrestrial pluvial in 2007 in North America (*33*–*36*) was propagated by an OWAS event that originated in the northeastern Pacific and migrated to the United States (Fig. 1A). Although there exist only 20.6% (436 events) of TWS pluvial events that are propagated by OWASs over the globe, with concentrations in regions such as NMHL (Fig. 1, B, E, H, and K), they are more persistent (+75%) and extensive (+48%) than the other TWS pluvial events (1678 events). These results suggest that the direct landward migration of oceanic water availability surpluses is more likely to trigger severe TWS pluvial events, especially in NMHL.

As the climate model simulations have considerable systematic biases relative to observations, bias correction methods are commonly recommended to correct these biases (*37*–*39*). Here, we used a quantile delta mapping (QDM) algorithm to correct biases in climate model simulations and projections to reduce the error in subsequent analysis (see Materials and Methods). We found that the spatial patterns of ERA5-based OWAS characteristics are relatively well captured by bias-corrected climate model simulations for CMIP6 multimodel mean during 1961–2020 [spatial correlations between historical climate (hereafter ALL) simulations and ERA5: 0.66 < *r* < 0.81; 0.04 < relative error < 0.39; *P* < 0.001; Fig. 1, D, G, and J]. This holds true for most OWAS characteristics across the CMIP6 individual model. High spatial correlations (*r* > 0.5; *P* < 0.001) are found in 93% of all combinations (119 of 128 combinations, i.e., 4 characteristics by 32 models) between ERA5 and CMIP6 individual models (fig. S2).

**Fig. 2.**
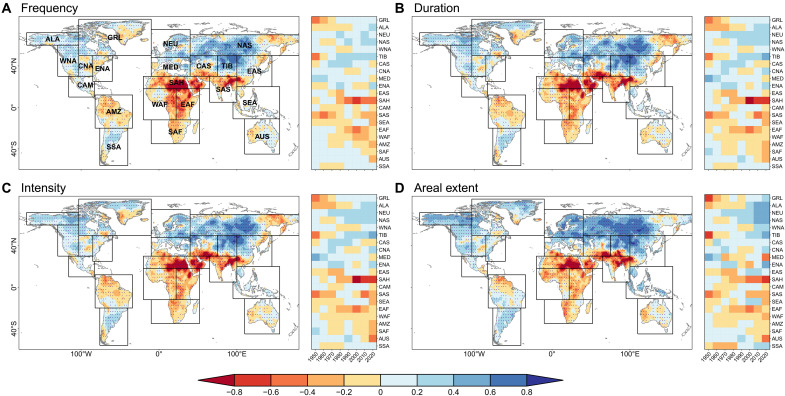
Human-induced relative changes in OWAS characteristics during 1921–2020. (**A** to **D**) Maps show the relative changes in frequency (A), duration (B), intensity (C), and areal extent (D) of OWASs based on CMIP6 under GHG effects over the globe during 1981–2010. Stippling indicates that more than 60% of the models agree on the changes in OWAS characteristics across CMIP6 models. Black boxes in maps include 21 different Giorgi climate regions. Heatmaps show regional relative changes in OWAS characteristics across 30-year moving windows from 1921 to 2020 under AER compared to under ALL in different climate regions. The label “2020” corresponds to the 1991–2020 window. GRL, Greenland and Northern Territories; ALA, Alaska; NEU, Northern Europe; NAS, North Asia; WNA, Western North America; TIB, Tibet; CAS, Central Asia; CNA, Central North America; MED, Mediterranean Basin; ENA, Eastern North America; EAS, East Asia; SAH, Sahara; CAM, Central America; SAS, South Asia; SEA, Southeast Asia; EAF, Eastern Africa; WAF, Western Africa; AMZ, Amazon Basin; SAF, Southern Africa; AUS, Australia; SSA, Southern South America.

Further, we investigated the response of OWAS characteristics to ANT, particularly greenhouse gas emissions (GHG) and anthropogenic aerosol emissions (AER). We attributed relative changes in OWAS characteristics to different external forcings compared to the historical climatological mean (1981–2010). Given that the OWAS characteristics under GHG closely align with those under ANT (fig. S3), we mainly focused on the OWAS response to GHG (Fig. 2). Spatially, positive changes in OWAS frequency, duration, intensity, and areal extent under CMIP6 GHG are found in 49.1, 50.6, 50.8, and 59.9% of the global land area during 1981–2010, respectively. Consistent (more than 60% model agreement) increases are found in NMHL, including five IPCC Giorgi regions. GHG-induced warming brings more moisture and a stronger land-sea thermal contrast (*20*, *40*), thereby creating favorable conditions for the landward migration of oceanic water availability surpluses. Temporally, relative changes in OWAS characteristics in NMHL under GHG transitioned from negative to positive values with enhancing GHG emissions beginning in the past two decades of the 20th century (Fig. 2).

### Migrations of historical OWASs in NMHL

**Fig. 3.**
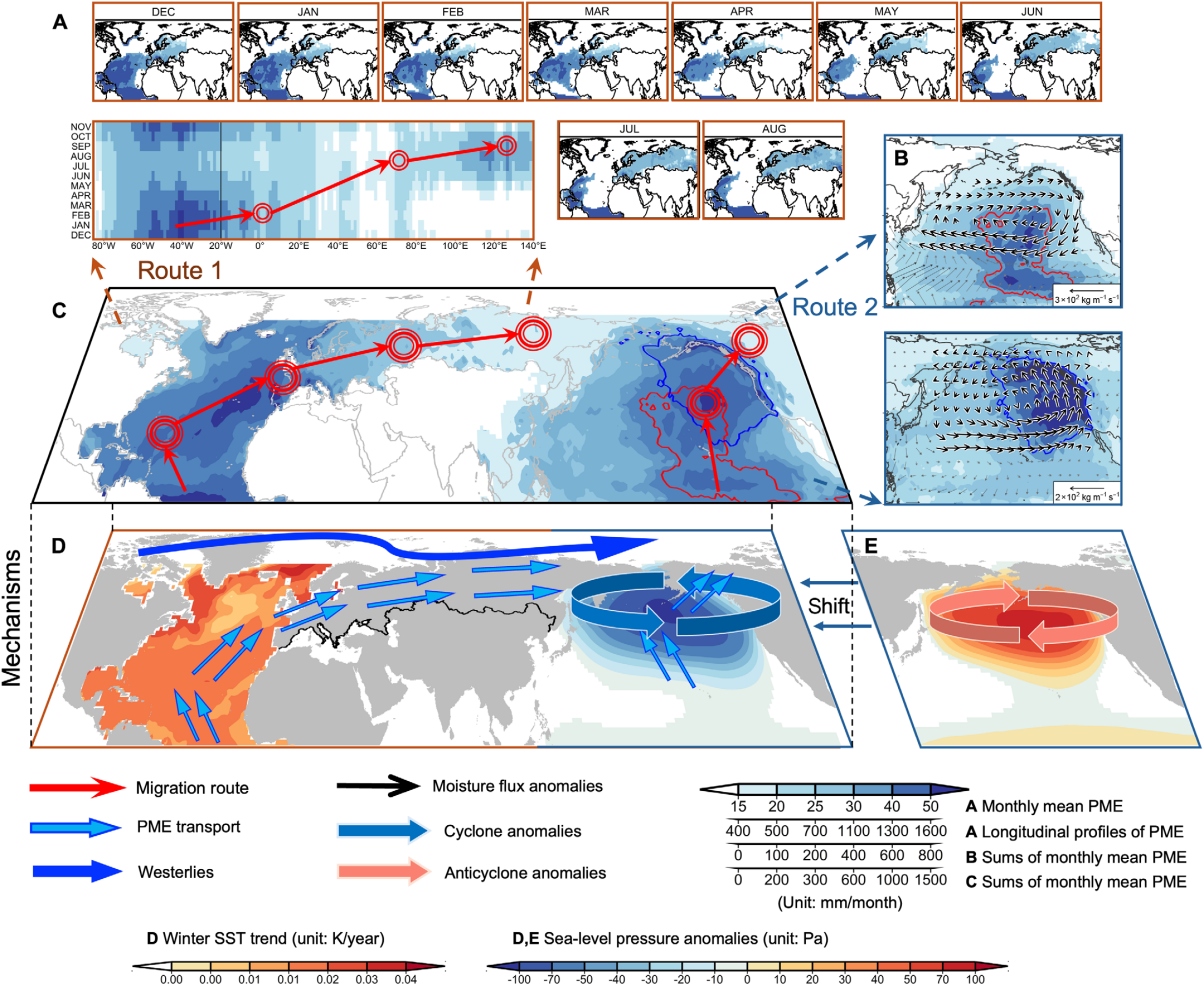
Landward migration routes and mechanisms of OWASs in NMHL from the northern Atlantic and northeastern Pacific. (**A**) Maps show water availability (PME) anomalies where the SWAI > 0.8 from December to August under CMIP6 ALL during 1961–2020. Heatmap shows longitudinal accumulation of monthly WAAs in the northern Atlantic and northern Eurasia. (**B**) Maps show composite anomalies of integrated moisture flux and cumulative WAAs over the northeastern Pacific and western North America between ALL during OWAS pre–land (top) or post–land (bottom) arrival period (1961–2020) and the climatological mean (1981–2010). The OWAS pre–land arrival period is the period from genesis to arrive at land. The OWAS post–land arrival period is the period from arriving land to extinction. (**C**) Map shows the cumulative WAAs from the northern Atlantic to northern Eurasia and from the northeastern Pacific to western North America during 1961–2020. Red and blue contours indicate areas where the cumulative WAAs over the northeastern Pacific exceed 500 mm month^−1^ during the pre–land arrival and post–land arrival period. (**D** and **E**) Maps show winter SST trends in the northern Atlantic during 1961–2020 (D), as well as composite sea level pressure anomalies in the northeastern Pacific during the pre–land arrival (E) and post–land arrival (D) period compared to the climatological mean, respectively.

Given that NMHL is a hotspot where OWASs tend to be stronger under ANT, we further elucidated the landward migration routes of OWASs. We identified two major migration routes of OWASs from the Atlantic and Pacific to NMHL during 1961–2020 (Fig. 3). The first route originates from the northern Atlantic and migrates to northern Eurasia through seasonal teleconnection (Fig. 3, A and C). Specifically, from December to February of the following year, water availability surpluses originate in the northwestern Atlantic and migrate northeastward to Europe. By March, the positive anomalies of water availability reach Europe and eventually arrive in northeastern Asia by August. The variations of sea surface temperature (SST) in the northern Atlantic play an important role in the landward migration of water availability surpluses along the first migration route (*41*–*43*), as indicated by a significantly positive correlation between SST and WAAs in the beginning season of this migration during 1961–2020 (*r* > 0.5; *P* < 0.05).

The second migration route of OWASs originates from the northeastern Pacific and migrates to western North America through the shift of circulation patterns (Fig. 3B). During the pre–land arrival period, northward migrating water availability surpluses are generated near the equator. However, these northward surpluses are impeded by anomalous high-pressure patterns over the northeastern Pacific (Fig. 3E). During the post–land arrival period, in contrast, the high-pressure patterns shift to low-pressure patterns over the northeastern Pacific (Fig. 3D). The shift in circulation patterns leads to the landward migration of water availability surpluses and their eventual arrival in western North America (Fig. 3C). This migration process could be influenced by SST in the Pacific. The warming tropical Pacific would cause the subtropical jet stream to extend eastward, resulting in anomalous cyclonic flows around a deepened Aleutian low pressure (*21*, *44*). These cyclonic flows drive large amounts of moisture north-eastward from the subtropical Pacific into western North America (*45*).

### Anthropogenically intensified OWASs in the future

**Fig. 4.**
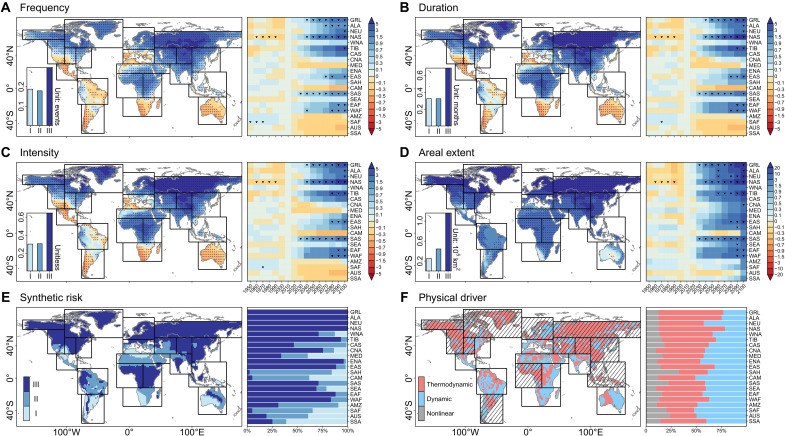
Projected relative changes in OWAS characteristics under SSP585. (**A** to **D**) Maps show projected relative changes in OWAS frequency (A), duration (B), intensity (C), and areal extent (D) between CMIP6 SSP585 during 2071–2100 and ALL during 1981–2010, respectively. Stippling indicates that more than 60% of the models agree on the changes in OWAS characteristics across CMIP6 models. Bar charts in maps [(A) to (D)] present OWAS characteristics for each risk level. Heatmaps accompanying the maps [(A) to (D)] show regional relative changes in 30-year moving windows under ALL + SSP585 during 1921–2100 compared to ALL during 1981–2010 in different climate regions, with “2100” representing the window of 2071–2100. Symbol “▼” indicates climate regions where the |SNR| > 1. (**E**) Map shows OWAS risk levels globally from CMIP6 SSP585 during 2071–2100. Bar chart represents the proportion of each climate region in different OWAS risk levels. (**F**) Thermodynamic, dynamic, and nonlinear components of normalized differences in moisture flux convergence (MFC) during OWASs between CMIP6 SSP585 during 2071–2100 and ALL during 1981–2010. Map shows the dominant MFC component in each grid cell. Hatchings on the maps show regions where the thermodynamic component dominates. Bar chart shows the contribution of each component in a climate region.

After revealing two major migration routes of OWASs over NMHL, we projected future changes in OWAS characteristics under the SSP585 scenario that incorporate time-varying natural and anthropogenic drivers. Dominated by GHG concentrations by the late 21st century, these projections emphasize the primary role of GHG and represent the climate response to ANT (*46*). The global land surface is projected to experience more frequent (+111%), persistent (+181%), severe (+194%), and widespread (+1225%) OWASs during 2071–2100 under SSP585 (Fig. 4, A to D). Consistent (more than 60% model agreement) increases are found in 72.8 to 99.2% of the globe across the four types of OWAS characteristics. Since the beginning of the 21st century (2001–2030), 62% (13 of 21) of IPCC Giorgi regions show a relative increase in all OWAS characteristics, but only one region exhibits a detectable anthropogenic signal [|signal-to-noise ratio (SNR)| > 1] for four characteristics. By the end of the 21st century (2071–2100), these increases become more substantial in 71% (15 of 21) of IPCC Giorgi regions, with the anthropogenic signal emerging in nine regions. These nine regions cover the entire NMHL, which experiences the most substantial increases (+261 to +2988%) in the four types of OWAS characteristics. The OWAS risk evaluation further suggests the highest risk level in NMHL (Fig. 4E and Supplementary Text). Therefore, our findings consistently reveal that the highest frequency and risk of OWASs, along with the most substantial increase in OWAS characteristics, are projected to be found in NMHL.

In the first OWAS migration route to NMHL, historical OWASs over northern Eurasia were associated with winter warming SST in the northern Atlantic (1.7 × 10^−2^ K year^−1^; *P* < 0.001; Fig. 3). The future warming rate of winter SST (3.9 × 10^−2^ K year^−1^; *P* < 0.001; 2021–2100) is 2.3 times faster than the historical rate (fig. S4). In addition, both winter water availability surpluses in the northern Atlantic and March to August surpluses in northern Eurasia are projected to significantly increase. Relationships between winter SST, winter surpluses in the northern Atlantic, and subsequent March to August surpluses in northern Eurasia are also projected to be much tighter (both *r* > 0.9; *P* < 0.001). Along this migration route, not only increasing SST and water availability surpluses but also their stronger linkages would enhance this seasonal teleconnection between the northern Atlantic and northern Eurasia under ANT, resulting in more severe and widespread OWASs in the future.

In the second migration route, historical OWASs over western North America were driven by cyclonic anomalies and negative sea level pressure over the northeastern Pacific. These circulation patterns are caused by the shift from high-pressure patterns during the pre–land arrival period to low-pressure patterns during the post–land arrival period over the northeastern Pacific (Fig. 3). The difference of sea level pressure anomalies between pre–land and post–land arrival periods is projected to be enlarged under ANT (fig. S4), indicating that this shift would be enhanced in the future. The enhanced shift in circulation patterns would result in widespread landward expansion and strengthening of water availability surpluses over the northeastern Pacific and hence lead to more OWASs migrating to western North America.

The anthropogenically induced increase in OWAS can be explained by moisture flux convergence (MFC) intensified thermodynamically and dynamically (*47*–*49*). Our atmospheric moisture budget diagnosis based on CMIP6 shows the contributions of thermodynamic and dynamic components to the changes in integrated MFC (≈ precipitation minus evapotranspiration) during OWASs between SSP585 during 2071–2100 and ALL during 1981–2010 (Fig. 4F). The increase in water availability is attributable to the thermodynamic (dynamic) component in 54.7% (42.4%) of regions globally. It is worth noting that, in addition to mean-state thermodynamic and dynamic contributions, transient eddies on synoptic timescales can also influence both components of MFC (*49*). Previous research has shown that MFC from transient eddies can be comparable to the monthly mean flow in the Pacific for the second route (*50*). However, because of the limited availability of subdaily CMIP6 simulations, transient eddies were not included in our analysis. In NMHL, the thermodynamic component is the dominant driver (76.8% of NMHL areas). Notably, there is a clear spatial overlap between regions with robust anthropogenic signals in OWAS intensification and those dominated by thermodynamic components—59 to 62% of such regions coincide. Spatial correlations between relative changes in OWAS characteristics and the thermodynamic components are stronger (0.24 < *r* < 0.26, *P* < 0.05) than correlations with dynamic and nonlinear components ( −0.07 < *r* < 0.08) over regions with robust anthropogenic signals. These underscore the primary role of anthropogenic thermodynamic processes. This thermodynamic effect can be further attributed to convergence (advection) across 72.9% (27.1%) of the thermodynamic-dominant regions (fig. S5). Thermodynamically, anthropogenically induced increases in oceanic water availability from warming SSTs and rising atmospheric water holding capacity (*47*, *51*) facilitate the migration of water availability surpluses from ocean to land.

## DISCUSSION

OWAS is a contiguous, large-scale migration phenomenon of excess atmospheric freshwater from ocean to land, favoring to mitigate long-term droughts or, conversely, act as potential precursors to pluvials. OWASs ranging from seasonal to yearly timescales are identified by the extreme high water availability and last no less than 3 months. Past and projected future hotspots of OWASs are primarily located in NMHL (Figs. 2 and 4), with major routes from the tropical Atlantic to northern Eurasia and the northeastern Pacific to northwestern North America (Fig. 3). These migrations of water availability surpluses from ocean to land undergo oceanic evaporation, atmospheric moisture transport and precipitation, and land evapotranspiration. In this cascading process, moisture from land evapotranspiration also contributes to OWASs, yet quantifying its role within the OWAS migration remains challenging due to complex ocean-land-atmosphere interactions and moisture cycles (*8*, *52*). However, the Lagrangian trajectory method tracks individual air parcels and quantifies moisture uptake and release along their trajectories, thereby establishing a physically explicit link between oceanic evaporation and land-based precipitation (*7*, *53*), albeit at a higher computational cost (*54*). In contrast, our coherent feature tracking can identify the long-term, large-scale migration patterns of excess water availability with lower computational demand, offering a macroscopic perspective on seasonal water redistribution that may alleviate droughts or trigger pluvials. Integrating both methods offers complementary strengths for diagnosing anomalous water availability migration (*5*, *11*, *55*, *56*).

It is worth noting that excess water availability over the ocean can also result from synoptic to mesoscale systems, such as tropical cyclones (TCs) and atmospheric rivers (ARs) (*57*, *58*). However, instead of tracking individual storm events, our analysis captures the aggregated signal of water availability surpluses at seasonal to yearly timescales, providing a coherent representation of large-scale and long-term atmospheric water redistribution. Moreover, OWASs, TCs, and ARs fundamentally differ in their characteristics, migration, etc. OWASs in NMHL show minimal association with TCs, due to that: (i) Timescales: TCs are short-lived, occurring on daily to subseasonal timescales and are identified by hourly wind speeds. Their durations do not exceed 3 months, whereas OWASs persist for at least 3 months or longer. (ii) Migration patterns: TCs predominantly originate and develop in the tropics (*59*). Specifically, Atlantic TCs originating make landfall along the eastern coast of North America (*60*), while those from the western (eastern) Pacific affect the coasts of eastern Asia (western Mexico) (*61*, *62*). These localized paths contrast with the more extensive and persistent migration routes of OWASs. (iii) Precipitation contributions: TCs contribute minimally to NMHL precipitation (*59*). Their associated precipitation is insufficient to penetrate inland NMHL regions, and their contribution to annual precipitation in NMHL coastal areas remains below 10% [see figure 3 in (*59*)]. This underscores the limited role of TCs in driving NMHL pluvials.

OWASs in NMHL and ARs differ in four aspects: (i) Timescales: ARs are synoptic-scale phenomena lasting days to weeks. The longest duration of all these ARs is shorter than 40 days (*63*), whereas OWASs typically endure for at least 3 months and can extend beyond a year. (ii) Seasonality: ARs in NMHL exhibit distinct seasonal patterns, occurring from October to December along the west coast of North America and from September to October in Europe (*64*). By comparison, OWASs in NMHL span longer durations, such as Atlantic those to northern Eurasia developing from December through the following August (Fig. 3A). (iii) Migration patterns: ARs, although spanning thousands of kilometers, have more geographically constrained impacts. Atlantic-origin ARs primarily affect Europe and do not reach Siberia (*65*), while Pacific-origin ARs typically make landfall in central North America (below 48°N) (*66*). OWASs, however, follow broader and more persistent migration routes, penetrating deeper into continental regions such as northeastern Eurasia. (iv) Precipitation contributions: We identified landfalling ARs based on the ERA5-based dataset for atmospheric river analysis (EDARA) dataset (see Materials and Methods). Landfalling ARs contribute to more precipitation in eastern North America and East Asia but account for less than 10% of annual total NMHL precipitation over NMHL (fig. S6). This suggests a potential disconnect between OWAS-related excess water availability in the NMHL and the influence of landfalling ARs. We also examined the similarity between OWAS and subtropical highs. The distinct migration routes and spatial distributions—such as Atlantic OWAS being driven by northward and westerly winds rather than subtropical highs and Pacific OWAS occurring in northwestern North America while subtropical highs affect East Asia (*67*, *68*)—suggest a low similarity between the two.

We also identified spatiotemporally contiguous IVT (integrated vapor transport)–based events using the same approach as OWASs, because IVT is widely used to identify ARs and measure total transported water vapor to a location (*5*, *66*). Over the ocean, IVT-based events are primarily distributed in low latitudes (below 20°N), exhibiting zonally oriented, band-like structures resembling ARs (fig. S7). In contrast, OWASs display a distinct meridional pattern, migrating from low-latitude oceans toward the NMHL. Unlike OWASs, the characteristics of these IVT-based events in NMHL did not exhibit a pronounced positive response to GHG emissions (fig. S8). Instead, their frequency, duration, and intensity show widespread declines across the west coast of North America and inland Eurasia. During 2071–2100 under SSP585, IVT-based events globally decrease in frequency, with no substantial increases in duration or intensity, in stark contrast to the intensifying characteristics of OWASs (fig. S9). Notably, western North America experiences further reductions in duration and intensity of IVT-based event relative to historical climatology. These contrasting responses highlight the unique role of OWASs in shaping terrestrial water availability patterns in NMHL under climate change.

Although we have systematically investigated the spatiotemporal changes of OWAS characteristics, uncertainties may arise because of differences among datasets, including both model structural uncertainty and internal variability. To reduce structural uncertainty, we used the multimodel mean derived from 32 CMIP6 models. This multimodel mean shows higher spatial correlations with ERA5-based OWAS characteristics during 1961–2020 than all individual models (Fig. 1 and fig. S2), enhancing the robustness of our findings. We also acknowledged that internal variability arising from differences in initial conditions introduces additional uncertainty in our results. To account for this, we leveraged the Canadian Earth System Model version 5 (CanESM5) large ensemble (*69*), which includes 50 ensemble members that differ only in their initial conditions. Compared to the CMIP6 multimodel mean, the ensemble mean from CanESM5 exhibits stronger spatial correlations and smaller relative errors with ERA5-based OWAS characteristics (Fig. 1 and fig. S10), indicating that uncertainties due to internal variability are relatively minor. The spatial patterns of OWAS characteristics based on the first CanESM5 ensemble member are highly consistent with those from the ensemble mean (0.80 < *r* < 0.91; *P* < 0.01), suggesting that the influence of initial conditions is very limited. Moreover, when considering only the spread associated with internal variability, we find that anthropogenic signals in increase of OWAS characteristics tend to emerge earlier and across more regions in future projections (Fig. 4 and fig. S11). Besides climatological characteristics, the projected changes, the two migration routes, and the attribution results for OWAS based on CanESM5 are consistent with those based on CMIP6 and ERA5 (figs. S10 to S15). The overall consistency among CanESM5, CMIP6, and ERA5 further supports the robustness of our results and indicates that they are unlikely to be artifacts of dataset-specific biases or model-dependent structural differences.

There is mounting evidence that the frequency and intensity of pluvials have markedly increased in NMHL over the past several decades (*70*–*74*). Under a warmer future, more frequent, persistent, widespread, and severe OWASs are projected in NMHL, with 638.1 million people exposed to OWAS risk by the end of the 21st century under SSP585. This heightened exposure is projected to increase the likelihood of severe pluvials, resulting in substantial economic losses for affected populations (*75*, *76*). Anthropogenic climate change is driving the emergence of more frequent, persistent, widespread, and severe OWASs in NMHL, with crucial implications for our understanding of climate change and its impacts, as well as for the development of adaptation strategies.

## MATERIALS AND METHODS

### Reanalysis datasets

We identified spatiotemporally contiguous migration of OWASs using precipitation (unit: millimeters) and latent heat flux (unit: watts per square meter) from the state-of-the-art ERA5 (1961–2020) (*77*–*79*). The other monthly variables [SST (unit: kelvin), vertical integral of eastward and northward water vapor flux (unit: kilograms per meter per second), and sea level surface air pressure (unit: pascal)] for the period 1961–2020 were acquired from the ERA5 reanalysis data. We used two other reanalysis datasets, namely, the MERRA2 (1981–2020) (*80*), and the JRA55 (1961–2020) (*81*), to examine the sensitivity of historical spatial patterns of OWAS to different reanalysis datasets.

We used the latest TWS-reconstructed dataset to identify TWS pluvial events. The GTWS-MLrec dataset provides machine learning–reconstructed TWS estimates and is more reliable than previous TWS datasets (*82*). We used the three versions of GTWS-MLrec, i.e., the Jet Propulsion Laboratory of California Institute of Technology, the Center for Space Research at the University of Texas at Austin, and the Goddard Space Flight Center of NASA. All raw grid datasets were interpolated to 1.5° by 1.5° resolution by using bilinear mapping to ensure consistency in spatial resolution across datasets.

To detect the landfalling ARs during 1961–2020, we use the AR database—EDARA—developed by Mo (*83*) using the latest and most recognized AR detection technology (“mtARget-v3”) (*84*). This database provides the AR locations and annual AR-induced precipitation at a global scale, at a spatial resolution of 0.25° and a 6-hour time step (00:00, 06:00, 12:00, and 18:00 UTC), based on an IVT threshold, to which geometric conditions are added according to the coherence of AR structures using ERA5. Further details of the AR detection can be found in (*63*).

### Simulations

We used the CMIP6 model simulations (table S1) covering the historical period (1920–2014; “historical” of CMIP, ALL) and future high-emissions scenario (2015–2100; “ssp585” of Scenario Model Intercomparison Project, i.e., SSP585). The ALL simulations represent climate models forced by both anthropogenic and natural external forcings. To distinguish the impact of individual external forcings on OWASs, we used several external forcing experiments derived from the Detection and Attribution Model Intercomparison Project of CMIP6, e.g., historical natural (“hist-nat,” NAT), historical greenhouse-gas (“hist-GHG,” GHG), and historical anthropogenic-aerosol (“hist-aer,” AER) forcings (*85*). We selected the 32 CMIP6 models that outputted monthly precipitation (“pr,” unit: millimeters), latent heat flux (“hfls,” unit: watts per square meter), SST (“tos,” unit: kelvin), specific humidity (“hus,” unit: kilograms per kilogram; 19 layers), surface air pressure (“ps,” unit: pascal), sea level surface air pressure (“psl,” unit: pascal), meridional winds (“va,” unit: meters per second; 19 layers), and zonal winds (“ua,” unit: meters per second; 19 layers). The first ensemble member “r1i1p1f1” was used for each model to avoid the variability within each climate model ensemble. The corresponding historical and future simulations were merged for each model to obtain time series over the period 1921–2100 (*86*). We calculated evapotranspiration from latent heat flux which was available in more CMIP6 models than evapotranspiration (“evspsbl”) (*87*). The above CMIP6 outputs were all interpolated to a 1.5° by 1.5° spatial resolution.

We also used the latest single model initial-condition large ensemble from the Canadian Centre for Climate Modelling and Analysis, i.e., the CanESM5 (*69*). CanESM5 is used for evaluating internal climate variability and uncertainty impacts of initial conditions (*88*). Given CMIP6 comprises a combination of internal climate variability and model formulation differences (i.e., structural uncertainty), we used the CanESM5 with CMIP6 to further evaluate the robustness of our findings. The CanESM5 simulations provide a host of experiments including ALL, NAT, GHG, and AER forcings as well as the SSP585 scenario, for which we used 50, 50, 50, 30, and 50 ensemble members, respectively. The same variables as CMIP6 in each ensemble member were also interpolated to a 1.5° by 1.5° spatial resolution. In addition, we also used projected future population data obtained from the Inter-Sectoral Impact Model Intercomparison Project (ISIMIP) (*89*) to calculate the exposure to OWASs. These data are derived from ISIMIP under SSP5, which are considered to be consistent with the CMIP6 scenarios used in this study (*90*, *91*).

### Bias correction algorithm and significance test methods

To minimize systematic biases in climate model simulations of meteorological variables relative to the reanalysis dataset (*92*), we applied a bias correction algorithm (i.e., QDM) (*38*) to reduce these biases. This method preserves the physical realism and temporal variability inherent in climate model simulations while aligning their climatological magnitude with the reanalysis dataset, which has been extensively applied in previous studies (*37*, *39*). The bias correction was conducted for each model independently. Further details of the bias correction algorithm can be found in Supplementary Text.

We used three types of significance tests, i.e., the Pearson correlation test for the correlation coefficient of two temporal sequences or spatial patterns, the Kolmogorov-Smirnov test for the difference between the probability distributions of different types of event characteristics, and the Mann-Kendall test for the trend of a temporal sequence.

### Identification and characteristics of OWASs

We used a coherent feature-tracking approach to track the spatiotemporal evolution of OWASs based on reanalysis (1961–2020) and climate model simulations (1921–2100). Given our focus on seasonal migration of excess water availability, we tracked OWASs in space and time based on the 3-month average of WAAs (reference period: 1981–2010). Water availability is defined by the difference between precipitation and evapotranspiration, and evapotranspiration ( E ) is calculated by latent heat flux ( LE ), i.e., E=LE/λ , where λ  is the latent heat of vaporization (2.45 M·J·kg^−1^). Monthly water availability often exhibits highly skewed, non-Gaussian distributions, with distributional shapes varying by regional climate characteristics (*93*). Therefore, through a nonparametric kernel density estimation (*93*–*95*), a SWAI was calculated using a monthly WAA at a given grid cell and calendar month

SWAI=∫−∞WAAf^(x)dx=∫−∞WAA1nh∑k=1nK(x−xkh)dx (1)

where $\hat{f}\left(x\right)$ represents the probability density function at the given calendar month, and K(·) represents the kernel function with bandwidth h and sampling size n . The optimal selection of the bandwidth was calculated by using a cross-validation method separately for each grid cell and calendar month (*93*). Moreover, we used a median filtering method to improve the robustness of the spatial identification of SWAI clusters (*96*–*100*). Previous studies chose 0.2 as a high aridity condition for standard index to describe water availability deficits (*92*, *95*, *101*). Here, we chose a high humidity threshold, i.e., SWAI greater than 0.8, to represent water availability surplus conditions, which indicates “excess water availability” or “water availability surpluses” in this study. We also used 0.7 and 0.9 to test the sensitivity of OWASs to threshold, suggesting high spatial correlation (*r* > 0.77; *P* < 0.001) of spatial frequency of OWASs for threshold ranging from 0.7 to 0.9.

Spatiotemporally contiguous water availability surpluses are tracked on the basis of the SWAI. First, we identified cells with water availability surpluses and merged adjacent cells to identify spatially contiguous (connected) clusters. Then, any spatially contiguous clusters that share an overlap area over consecutive time steps were consolidated into a single spatiotemporal cluster. The spatiotemporally contiguous water availability surpluses are required to last at least 3 months. Spatiotemporally contiguous surplus events that originate entirely over oceans and then spread to land over an area of at least 100,000 km^2^ are identified as OWASs. In addition, we performed sensitivity tests for connection area and overlap area. Given that the number of OWASs is not sensitive to the selection of connection area, we chose a connection area threshold of 10,000 km^2^, as in previous studies with three-dimensional clusters (*92*). A total of 100,000 km^2^ is selected as overlap area threshold because the number of OWASs starts to converge at this value for different connection areas.

The characteristics were extracted from all identified OWASs: the duration (unit: months), defined as the lifetime of an OWAS; the maximum area (unit: square kilometers), the spatial extent of all land grid cells of an OWAS; and the intensity (unitless), the area-weight average SWAI of all land grid cells of an OWAS. We also mapped these characteristics at each grid cell to assess the spatial patterns of OWASs.

In addition, we standardized TWS anomalies from GTWS-MLrec and identified spatiotemporally contiguous TWS pluvial events in the same way as SWAI and spatiotemporally contiguous water availability surplus, respectively. The propagation from OWAS to TWS-based event is considered as a terrestrial pluvial event that starts during an OWAS with an overlap area of at least 10,000 km^2^ between two events according to certain spatiotemporal overlap rules (*31*, *32*). We identified OWAS-propagated TWS pluvial events from three version of GTWS-MLrec and calculated their spatial mapping average in this study.

### Anthropogenic influence on OWAS characteristics

We calculated the relative changes in OWAS characteristics ( I ) between given periods ( P ) in different external forcing ( F ; NAT, GHG, and AER) compared to historical climate (ALL), as follows

$$\begin{array}{c} \Delta I_{P,F}=\frac{I_{\text{ALL},P}-I_{F,P}}{I_{\text{ALL},P}}\times 100\% \end{array}$$ (2)

Following previous studies (*102*–*104*), we considered relative changes between ALL and NAT as ANT effects, between ALL and AER as GHG effects, etc. Similarly, in a future warmer climate, the projected relative changes were calculated as

$$\begin{array}{c} \Delta I_{P,\text{future}}=\frac{I_{\text{SSP}585,P}-I_{\text{ALL},1981-2010}}{I_{\text{ALL},1981-2010}}\times 100\% \end{array}$$ (3)

We also calculated the SNR to assess the relative contribution of the internal climate variability and the forced response under external forcings (*12*, *46*), as

SNR=ΔIP,F¯σ(ΔIP,F) (4)

where the signal is $\bar{{\Delta I}_{P,F}}$ , the forced response which is the characteristics of OWASs based on CMIP6 multimodel mean (CanESM5 ensemble mean). The noise is defined as $\sigma \left({\Delta I}_{P,F}\right)$ , the intermodel or intermember SD of the relative change in OWAS characteristics. For CMIP6, the signal and noise reflect both model structural uncertainty and internal variability across different models, whereas for CanESM5, they primarily represent internal variability derived from multiple ensemble members within a single model framework. The absolute value of SNR greater than 1 (less than 1) implies that the effect of external forcing is stronger (weaker) than that of combined uncertainties of internal climate variability and model formulation differences in CMIP6 (internal climate variability in CanESM5), i.e., the anthropogenic signals does (does not) emerge (*105*).

### Atmospheric moisture budget diagnosis

The atmospheric moisture budget diagnosis asserts that water availability equals the integrated MFC (*5*, *87*, *106*)

MFC=−1ρg∫ptps∇·(Vq)dp=P−E (5)

where ∇· is the divergence operator, ρ is the water density, g is the gravitational acceleration, $p_{t}$ is the pressure at the top of the atmosphere, $p_{s}$ is the near-surface pressure, q is the specific humidity, and V is the mean wind field (meridional wind v and zonal wind u ). MFC is approximately estimated as

MFC=−1ρg∫ptps∇·(V¯q¯)dp+transient (6)

where $\bar{V}$ is the monthly mean wind field, $\bar{q}$ is the monthly mean specific humidity, and transient presents MFC from transient eddies on intraseasonal or submonthly timescales (*5*, *49*), which are not included in the analysis due to the unavailability of subdaily simulations. The changes in MFC between future and historical periods can be decomposed as (*87*, *107*)

$$\begin{array}{c} {\text{MFC}}_{f}-{\text{MFC}}_{h}=-\frac{1}{\rho g}\int_{p_{t}}^{p_{s}}\left[\nabla \cdot \left({\bar{V}}_{f}{\bar{q}}_{f}\right)-\nabla \cdot \left({\bar{V}}_{h}{\bar{q}}_{h}\right)\right]\mathrm{dp}+H\\ =\underset{\text{Thermodynamic}}{\underbrace{-\frac{1}{\rho g}\int_{p_{t}}^{p_{s}}\nabla \cdot \left[{\bar{V}}_{h}\left({\bar{q}}_{f}-{\bar{q}}_{h}\right)\right]\mathrm{dp}}}\underset{\text{Dynamic}}{\underbrace{-\frac{1}{\rho g}\int_{p_{t}}^{p_{s}}\nabla \cdot \left[\left({\bar{V}}_{f}-{\bar{V}}_{h}\right){\bar{q}}_{h}\right]\mathrm{dp}}}\\ \underset{\text{Nonlinear}}{\underbrace{-\frac{1}{\rho g}\int_{p_{t}}^{p_{s}}\nabla \cdot \left[\left({\bar{V}}_{f}-{\bar{V}}_{h}\right)\left({\bar{q}}_{f}-{\bar{q}}_{h}\right)\right]\mathrm{dp}}}+H \end{array}$$ (7)

where the subscript f and h respectively indicate the future period of 2071–2100 under SSP585 and historical period of 1981–2010 under ALL, e.g., ${\text{MFC}}_{f}=-\frac{1}{\rho g}\int_{p_{t}}^{p_{s}}\nabla \cdot \left({\bar{V}}_{f}{\bar{q}}_{f}\right)\mathrm{dp}$ and ${\text{MFC}}_{h}=$$-\frac{1}{\rho g}\int_{p_{t}}^{p_{s}}\nabla \cdot \left({\bar{V}}_{h}{\bar{q}}_{h}\right)\mathrm{dp}$ respectively are the integrated MFC during the life span of OWASs from 2071 to 2100 and from 1981 to 2010. The dynamic component represents changes in horizontal wind, the thermodynamic component represents changes in specific humidity, and the nonlinear component represents changes in specific humidity and wind (*5*, *48*, *87*, *106*). H denotes higher-order term from contributions in transient eddies. In addition, the MFC can be composed of advection component and convergence component (*5*, *71*, *108*)

MFC=−1ρg∫ptpsV∇qdp⏟Advection−1ρg∫ptpsq∇·Vdp⏟Convergence (8)

The advection component indicates the horizontal advection of specific humidity, and the convergence component indicates the product of specific humidity and horizontal mass convergence (*5*, *49*). Further, the changes in MFC for Eq. 7 can be decomposed into multiple terms as follows (*92*)

$$\begin{array}{c} {\text{MFC}}_{f}-{\text{MFC}}_{h}\\ =\underset{\text{Advection Thermodynamic}}{\underbrace{-\frac{1}{\rho g}\int_{p_{t}}^{p_{s}}{\bar{V}}_{h}\nabla \left({\bar{q}}_{f}-{\bar{q}}_{h}\right)\mathrm{dp}}}\underset{\text{Convergence Thermodynamic}}{\underbrace{-\frac{1}{\rho g}\int_{p_{t}}^{p_{s}}\left({\bar{q}}_{f}-{\bar{q}}_{h}\right)\nabla \cdot {\bar{V}}_{h}\mathrm{dp}}}\\ \underset{\text{Advection Dynamic}}{\underbrace{-\frac{1}{\rho g}\int_{p_{t}}^{p_{s}}\left({\bar{V}}_{f}-{\bar{V}}_{h}\right)\nabla {\bar{q}}_{h}\mathrm{dp}}}\underset{\text{Convergence Dynamic}}{\underbrace{-\frac{1}{\rho g}\int_{p_{t}}^{p_{s}}{\bar{q}}_{h}\nabla \cdot \left({\bar{V}}_{f}-{\bar{V}}_{h}\right)\mathrm{dp}}}\\ \underset{\text{Nonlinear}}{\underbrace{-\frac{1}{\rho g}\int_{p_{t}}^{p_{s}}\nabla \cdot \left[\left({\bar{V}}_{f}-{\bar{V}}_{h}\right)\left({\bar{q}}_{f}-{\bar{q}}_{h}\right)\right]\mathrm{dp}}}+H \end{array}$$ (9)

### Subcontinental Giorgi regions

We used Giorgi climate regions, an IPCC climate reference regions (*28*), namely, Greenland and Northern Territories, Alaska, Northern Europe, North Asia, Western North America, Tibet, Central Asia, Central North America, Mediterranean Basin, Eastern North America, East Asia, Sahara, Central America, South Asia, Southeast Asia, Eastern Africa, Western Africa, Amazon Basin, Southern Africa, Australia, and Southern South America. The results for the globe and different IPCC Giorgi climate regions were area-weighted averages.

### Comparison with OWAS and AR

On the basis of EDARA, we identified landfalling ARs by whether it is solely a terrestrial source or not. These landfalling ARs originating from oceans account for 32.8% (14,530 of 44,280) of total AR events. We further quantified the contributions of AR-induced precipitation to annual total precipitation based on EDARA and ERA5 (fig. S6). In addition, we also identified IVT-based events and quantified their characteristics in ERA5 and CMIP6 (figs. S7 to S9), applying the AR definition (IVT exceeding a specified threshold) and the OWAS identification criteria. IVT, calculated by combining specific humidity and wind field from surface to top of the atmosphere, is widely used to identify AR events (*66*) and to quantify total transported water vapor to a location (*5*). The IVT-based events not only reflect the information of AR events but also provide a viewpoint of atmospheric moisture transport. This allowed us to investigate whether the OWASs identified by water availability are different with both ARs and IVT.

## Supplementary Materials

### This PDF file includes:

Supplementary Text Figs. S1 to S15 Table S1 References
