# Supplementary material for: Excess water availability in northern mid-high latitudes contiguously migrated from ocean under climate change
Source: Sci Adv. 2025 Aug 13;11(33):eadv0282. doi: 10.1126/sciadv.adv0282 (PMC12346308; doi:10.1126/sciadv.adv0282)
Supplement: Supplementary file 1 — Supplementary Text Figs. S1 to S15 Table S1 References [file sciadv.adv0282_sm.pdf]

Science Advances
AAAS

Supplementary Materials for

Excess water availability in northern mid-high latitudes contiguously migrated from ocean under climate change

Yansong Guan *et al.*

Corresponding author: Xihui Gu, guxh@cug.edu.cn

Sci. Adv. **11**, eadv0282 (2025)
DOI: 10.1126/sciadv.adv0282

This PDF file includes:

Supplementary Text
Figs. S1 to S15
Table S1
References

Supplementary Text

Synthetic risk evaluation of OWASs

To synthesize different aspects of OWAS characteristics (time, space, and intensity) for identifying regions that require special management attention, we employed an integrated machine learning method, the SOM-Kmeans clustering model (*112*). This model integrates the advantages of two cluster approach: self-organizing map (SOM) and Kmeans. SOM algorithm is a practical classification framework based on neural network for extreme weather and climate (*113–116*). SOM generates a feature matrix ($m \times n$) with the best architecture achieved by minimizing the efficiency index (*117*). Given the two-dimensional mapping of the matrix of SOM, a larger matrix gives a finer and more accurate regional classification, but this also generates more groups (*118*). Hence, we used the K-means algorithm to coarsen the feature matrix to produce a smaller number of groups on the SOM-based dissimilarity matrix (*119*).

Here, we selected the frequency, duration, intensity, and areal extent of OWASs under historical and future 30-year climatological periods, namely the historical ALL simulations during 1981–2010 and the SSP585 projections during 2071–2100. We produced a matrix (the number of land grid cells × 4 metrics) to input SOM. The best SOM dimension (the number and visual structure of the output neurons in SOM) is determined by the minimum values of the quantization error (QE) and the topological pattern error (TE) (*117*). QE is used to estimate the capacity of the framework of neural network to distinguish the input data, and TE is used to estimate whether the network is robust for classification task. In addition, the best cluster groups in Kmeans are determined by the minimum value of Davies-Bouldin index (DBI) (*120*). After data standardization, we calculated the QE, TE, and DBI to establish the best framework ($3 \times 2 = 6$ nodes in SOM, 3 groups in Kmeans) of SOM-Kmeans.

By employing SOM-Kmeans best framework, past and future risks of OWASs are categorized from low-level I to high-level III risk (Fig. 4E). During 2071–2100, areas with high-level risk are projected to experience extreme OWASs with a frequency of 3.0 events·decade^{-1}; duration of 6.9 months·decade^{-1}; intensity of 6.5 decade^{-1}; areal extent of 11.6·10^{5} km^{2}·decade^{-1}. Globally, high-level risk that did not exist in the historical climatological period, is projected to emerge in 61.1% of land areas under SSP585. Regionally, 62% of IPCC Giorgi regions (covering the entire NMHL) have more than half of their area at the high-level risk.

Quantile delta mapping bias correction algorithm

Given the substantial systematic biases in climate model simulations compared to observations and reanalysis datasets, we applied a bias correction algorithm, i.e., quantile delta mapping (QDM), to correct these biases. QDM is an integrated approach that combines quantile delta change (*121*), quantile perturbation (*122*), and detrended quantile mapping methods (*123*). The detrended annual time series are used to build the quantile mapping between simulations and reanalysis. Then the trends in the simulations are recovered by multiplying the bias-corrected simulations with the trends in the uncorrected simulations, to effectively reduce systematic biases and simultaneously preserve the relative change in simulations (*38*). First, the time series are detrended and then rearranged by scaling and rescaling.

$$\hat{x}_{m,p}(t) = F_{r,h}^{-1}\left\{F_{m,h}\left[\frac{\bar{x}_{m,h} \cdot x_{m,p}(t)}{\bar{x}_{m,p}(t)}\right]\right\} \cdot \frac{\bar{x}_{m,p}(t)}{\bar{x}_{m,h}} \tag{10}$$

where $\bar{x}_{m,h}$ is the mean value of simulation during the historical period and the $\bar{x}_{m,p}(t)$ is the projected period at the time of *t*. The $F_{r,h}$ and $F_{m,h}$ are the cumulative distribution functions (CDFs) of reanalysis-based variable $x_{r,h}$ and simulated variable $x_{m,h}$ in the historical period, respectively. Further, the bias in the non-exceedance probability quantile ($\tau_{m,p}$) at time *t* is corrected by using the inverse CDF of reanalysis during the historical period ($x_{r,h}$).

$$\hat{x}_{r:m,h:p}(t) = F_{r,h}^{-1}\left[\tau_{m,p}(t)\right] \tag{11}$$

Finally, the projection quantiles changes are transferred to the bias-corrected simulations, by using the relative change $\Delta_m(t)$ multiplied by its historical bias-corrected value (*38*).

$$\hat{x}_{m,p}(t) = \frac{\hat{x}_{r:m,h:p}(t) \cdot x_{m,p}(t)}{F_{m,h}^{-1}\left[\tau_{m,p}(t)\right]} \tag{12}$$

The dominant source of uncertainty in the climate model simulations comes from the model structure, which is larger than that of the scenario and ensemble (*124*). As a result, the outputs from a specific model can share similar biases, even under different forcing experiments (*39*). Therefore, QDM is first applied to correct the CDFs of ALL experiment to the ERA5-based CDFs; and then the same CDFs of ALL simulations are applied to correct the simulations under different forcings (NAT, AER, and GHG) and the projections (SSP585) (*39*). The bias correction was conducted for each model independently.

Supplementary Figures

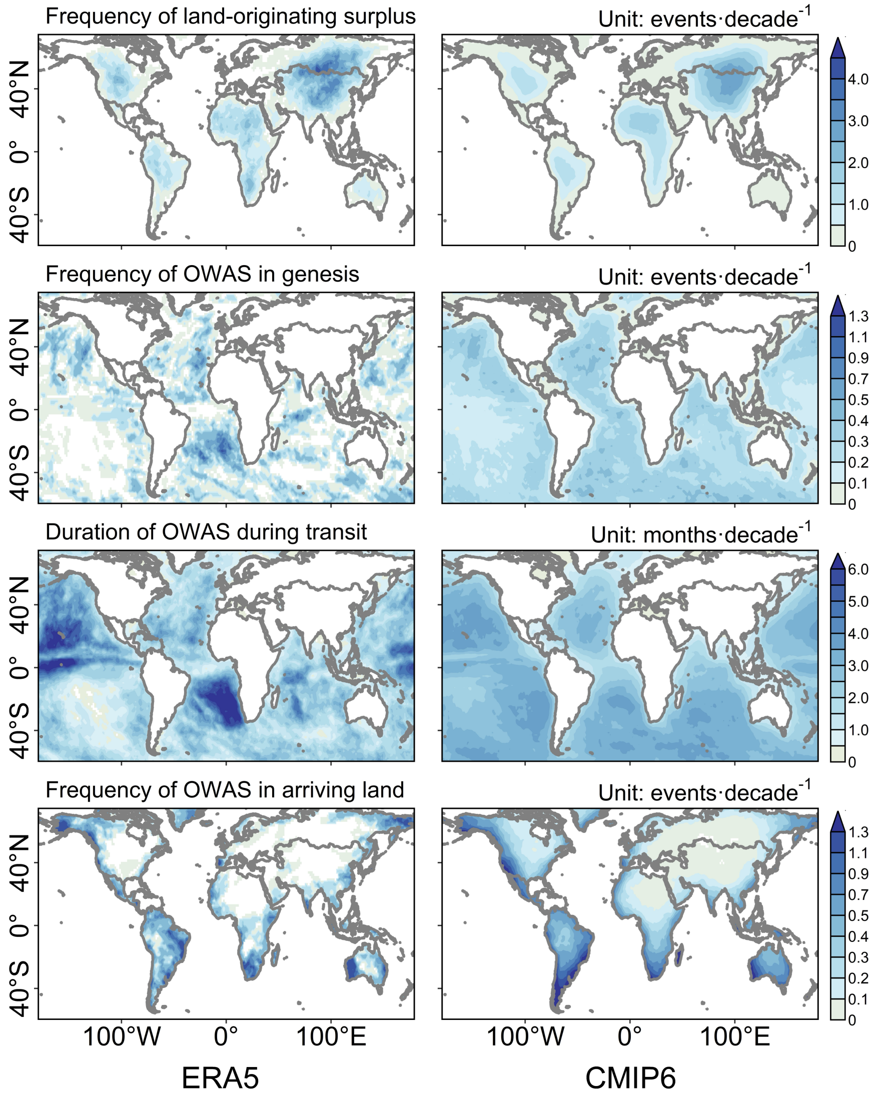

Fig. S1. Frequency of land-originating and ocean-to-land water availability surpluses (OWAS). Maps show the frequency of land-originating surpluses and that of OWASs in the genesis, transit, and arriving land periods based on ERA5 (left column) and CMIP6 (right column) over the globe during 1961–2020. The genesis indicates the initial moment in occurrence of each event; the transit indicates a period from initial moment until arriving land; the arriving land refers to the initial moment when an OWAS event first covers a land area more than 100,000 km^2.

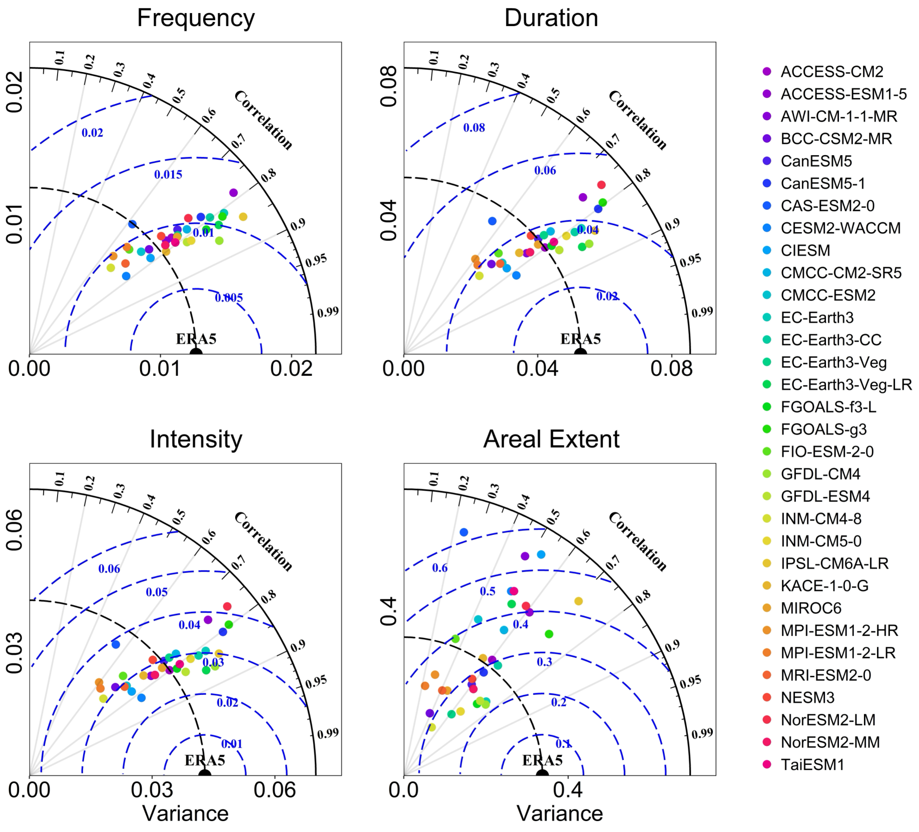

Fig. S2. Comparison of climatological characteristics of OWAS during 1961–2020 between CMIP6 models and ERA5. Taylor diagrams show the correlation of frequency, duration, intensity, and areal extent of OWASs during 1961–2020 between CMIP6 individual model and ERA5.

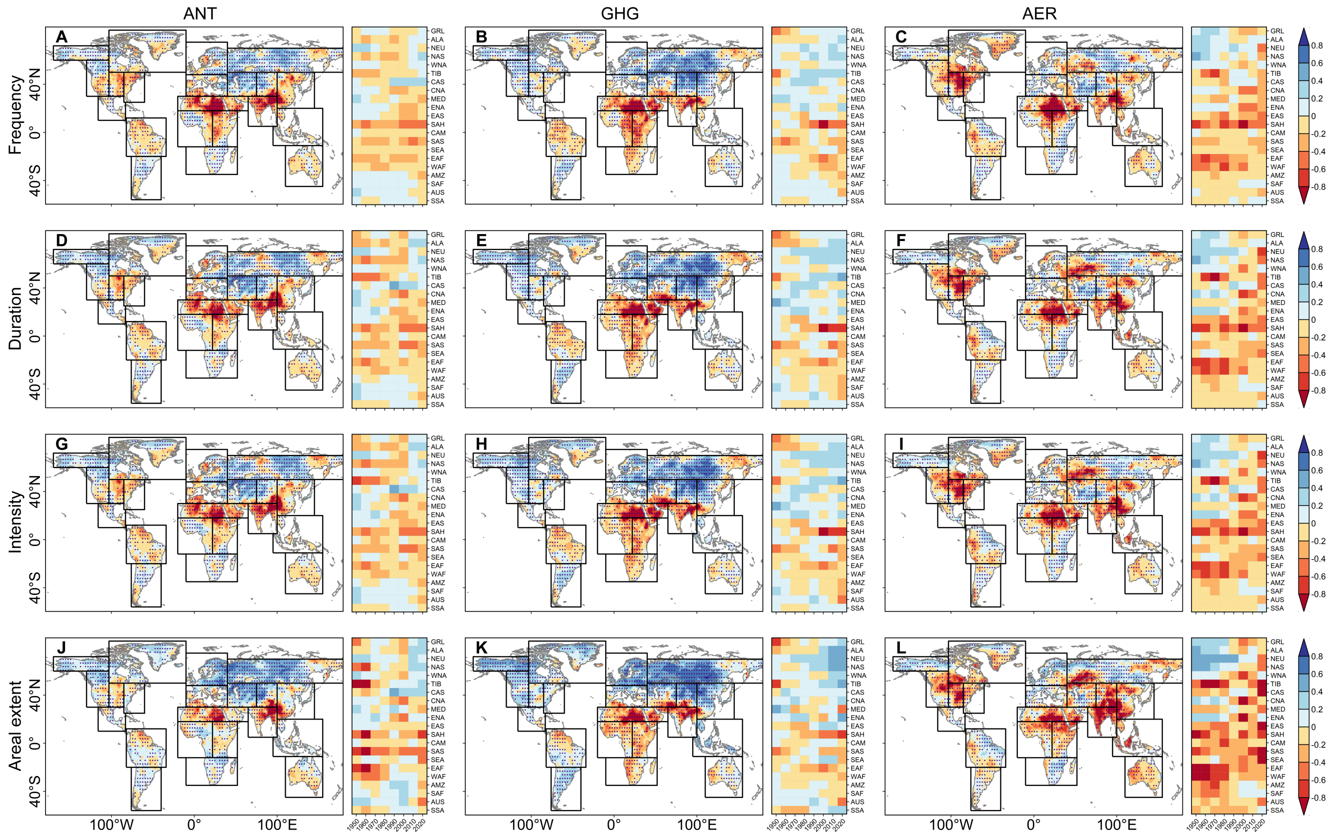

Fig. S3. Human-induced relative changes in OWAS characteristics during 1921–2020. (A)–(L), Maps show the relative changes in frequency from (A)–(C), duration from (D)–(F), intensity from (G)–(I), and areal extent from (J)–(L) of OWASs based on CMIP6-EM under ANT effects, GHG effects, and AER effects over the globe during 1981–2010. Stipples show that more than 60% of the models agree on the changes in OWAS characteristics across CMIP6 models. Black boxes in maps include 21 different Giorgi climate regions. Heatmaps show regional relative changes in 30-year moving windows during 1921–2020 under different external forcings (NAT, GHG, and AER) compared to under ALL in different Giorgi climate regions. The label "2020" corresponds to the 1991–2020 window.

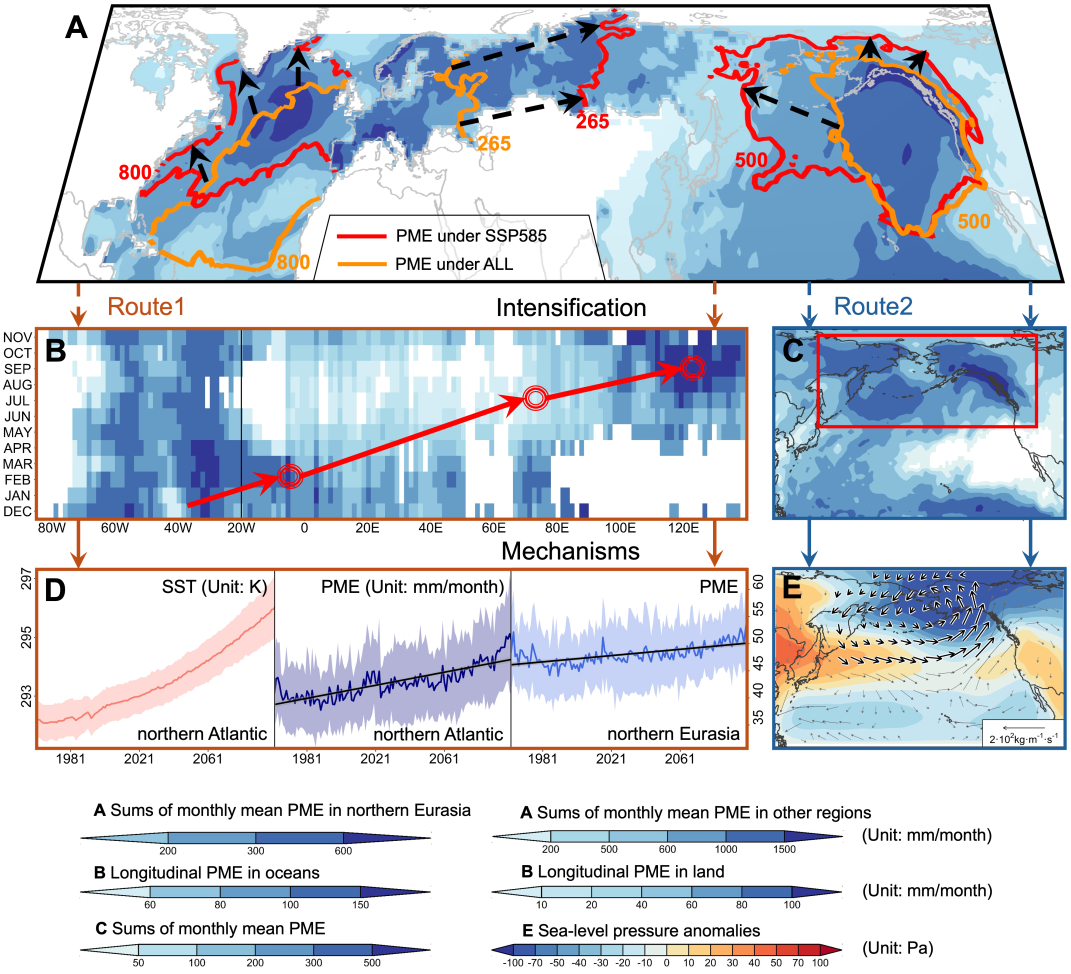

Fig. S4. Projected future migration routes and mechanisms of OWASs in NMHL from the northern Atlantic and northeastern Pacific. (**A**) Map shows the cumulative water availability (PME) anomalies of OWASs migrating to northern Eurasia from the northern Atlantic and western North America from the northeastern Pacific during 2021–2100 under CMIP6 SSP585. Orange (red) contours indicate areas where the cumulative water availability of OWASs over northern Atlantic, northern Eurasia, and northeastern Pacific exceeds 800, 265, and 500 mm·month^{-1} under ALL during 1961–2020 (under SSP585 during 2021–2100), respectively. (**B**) Heatmap shows changes in longitudinal accumulation of monthly mean water availability anomalies in the northern Atlantic and northern Eurasia under SSP585 during 2021–2100 compared to ALL during 1961–2020. (**C**) Maps show changes in cumulative water availability anomalies of OWASs over the northeastern Pacific and western North America during the post-land arrival period of OWASs under SSP585 during 2021–2100 compared to ALL during 1961–2020. (**D**) Curve lines show temporal changes in area-weighted average winter SST in the northern Atlantic, winter water availability in the northern Atlantic, and water availability from March to August in northern Eurasia during 1961–2100 under ALL+SSP585. Shading represents the standard deviation of those across the CMIP6 multi-model ensemble. (**E**) Maps show changes in difference of integrated moisture flux and sea-level pressure anomalies in the northeastern Pacific between during post-land arrival and pre-land arrival period of OWASs under SSP585 during 2021–2100 compared to ALL during 1961–2020.

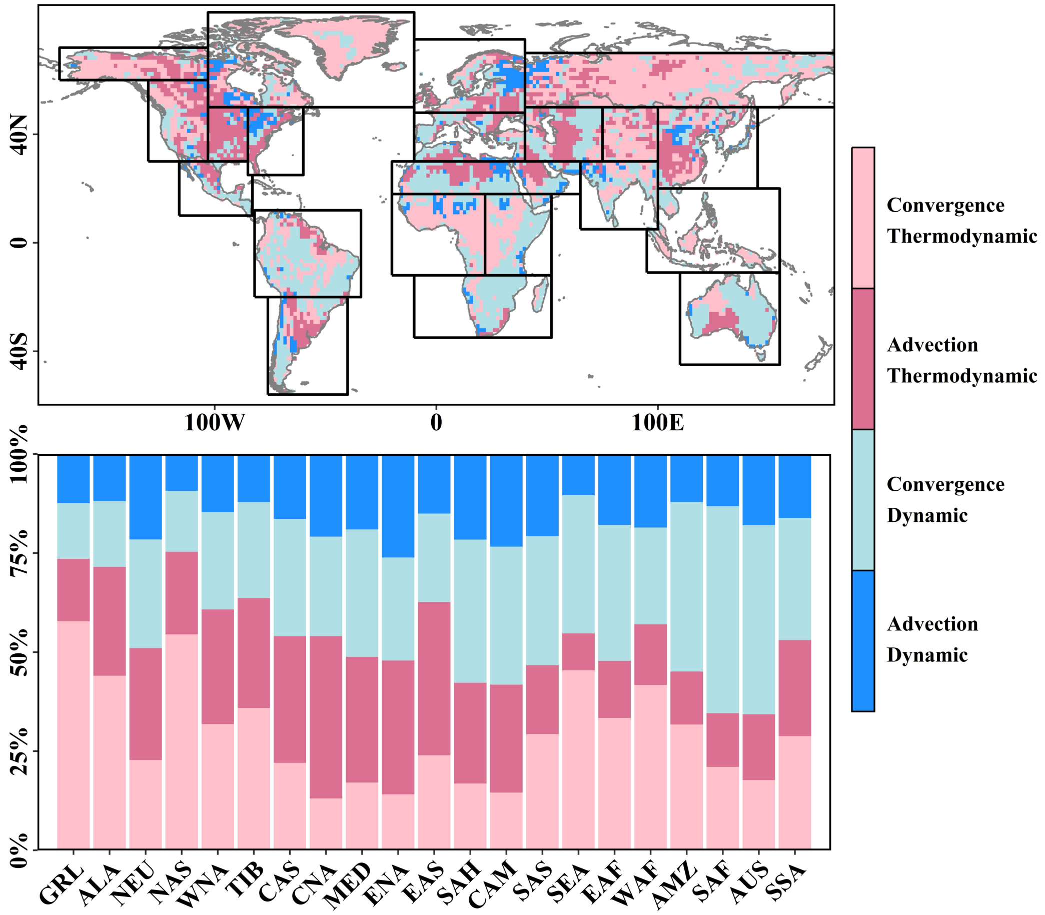

Fig. S5. Contributions of convergence and advection components to moisture flux convergence during OWASs under SSP585. Map shows the contributions of four components across each grid cell for convergence thermodynamic, convergence dynamic, advection thermodynamic, and advection dynamic components of moisture flux convergence during the lifespan of OWASs between SSP585 during 2071–2100 and ALL during 1981–2010 based on CMIP6 in different Giorgi climate regions. Bar chart shows the contribution ratios of four components in different Giorgi climate regions.

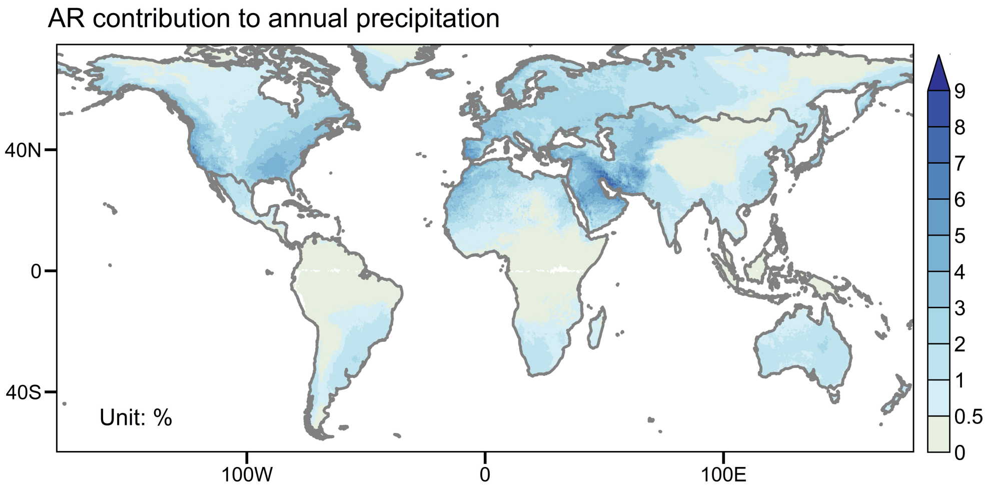

Fig. S6. Contribution of landfalling atmospheric river (AR) to annual precipitation. Map show the relative contribution (%) of AR precipitation to the annual total precipitation based on EDARA and ERA5.

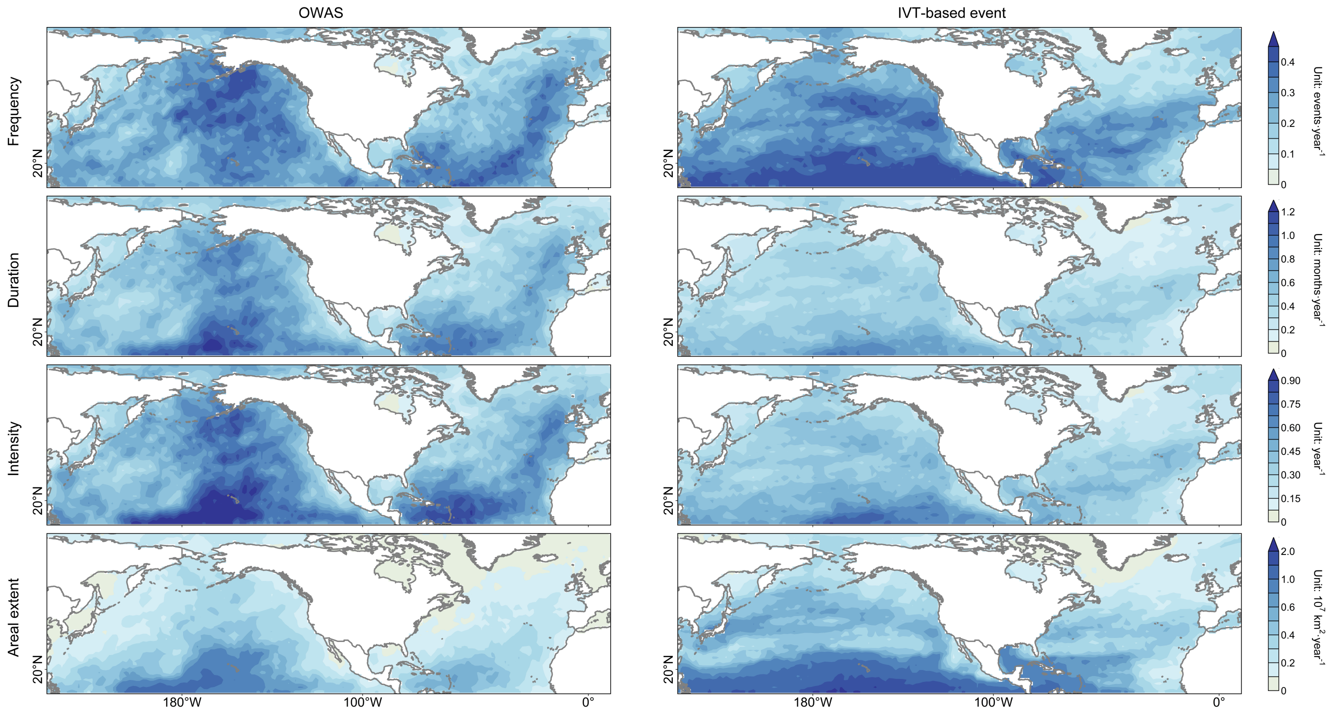

Fig. S7. Climatological characteristics of OWAS and IVT-based event during 1961–2020. Maps show the frequency, duration, intensity, and areal extent of OWASs and IVT-based events over the ocean based on ERA5 during 1961–2020.

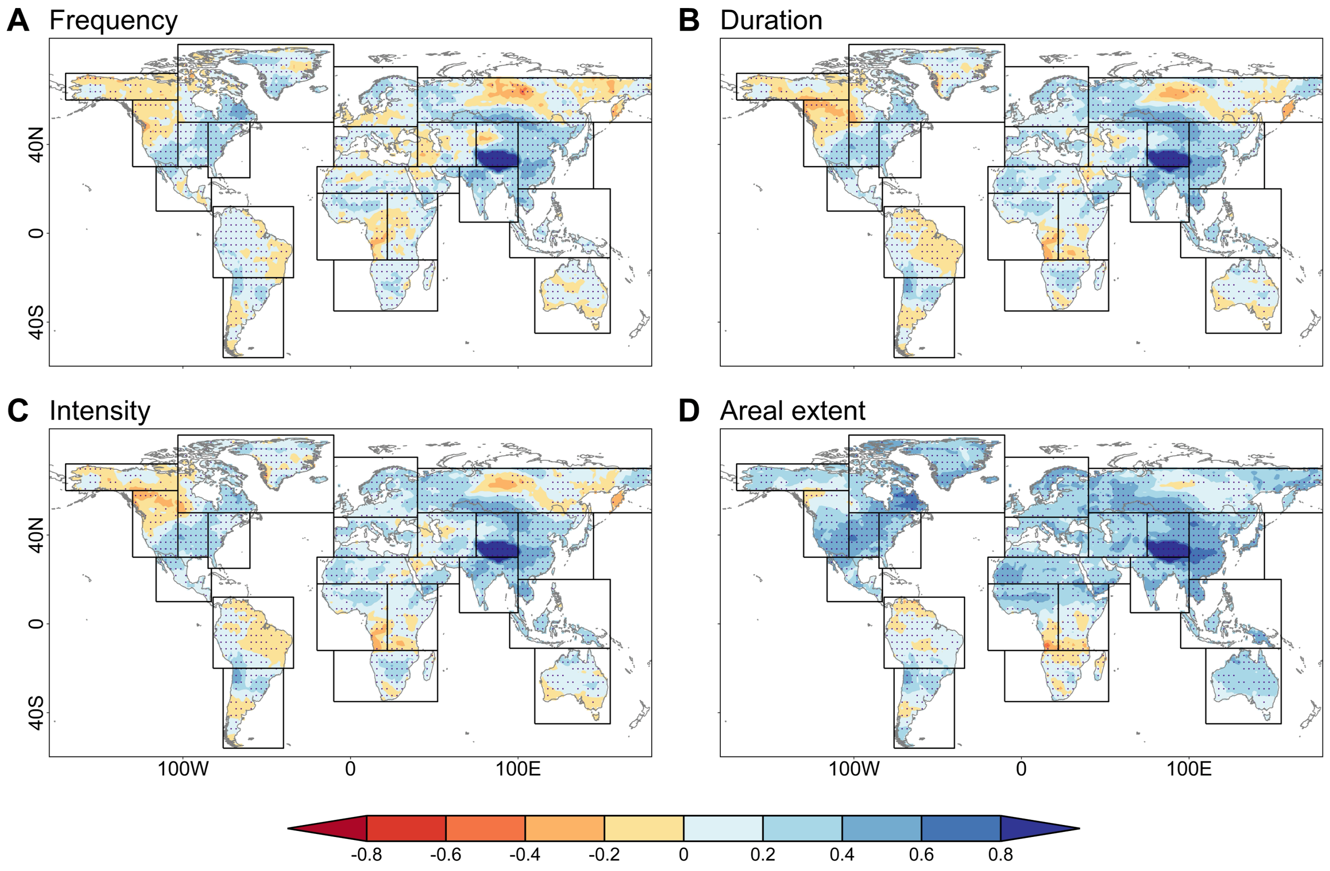

Fig. S8. Human-induced relative changes in IVT-based event characteristics during 1921–2020. (A)–(D) Maps show the relative changes in frequency (A), duration (B), intensity (C), and areal extent (D) of IVT-based events based on CMIP6 under GHG effects over the globe during 1981–2010. Stippling indicates that more than 60% of the models agree on the changes in OWAS characteristics across CMIP6 models. Black boxes in maps include 21 different Giorgi climate regions. Heatmaps show regional relative changes in characteristics of IVT-based events across 30-year moving windows from 1921 to 2020 under AER compared to under ALL in different climate regions. The label “2020” corresponds to the 1991–2020 window.

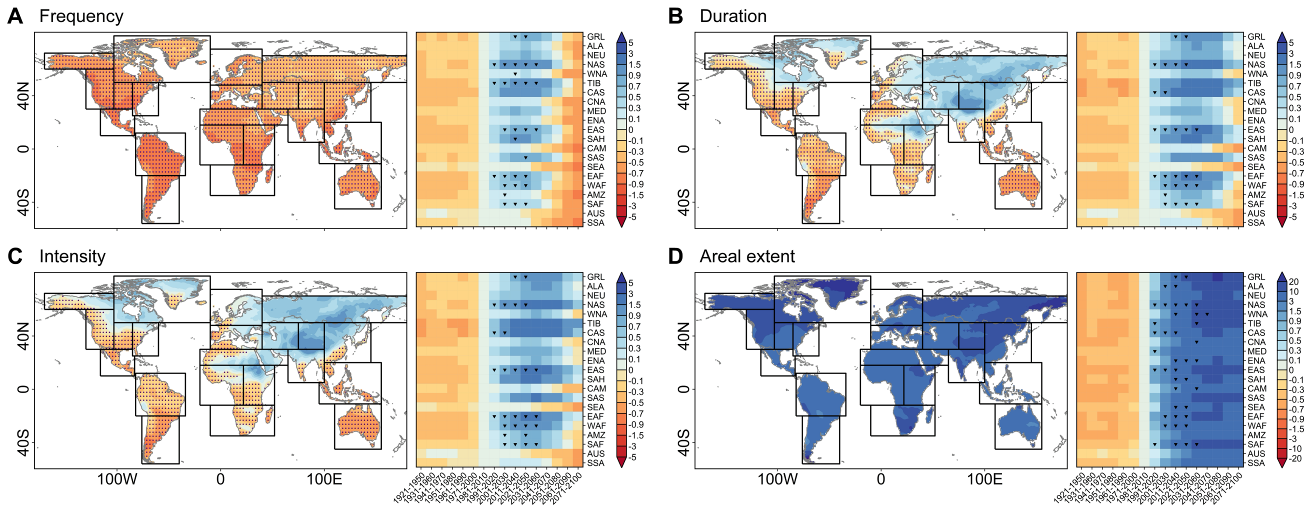

Fig. S9. Projected relative changes in IVT-based event characteristics under SSP585. (**A**)–(**D**) Maps show projected relative changes in frequency (A), duration (B), intensity (C), and areal extent (D) of IVT-based events between CMIP6 SSP585 during 2071–2100 and ALL during 1981–2010, respectively. Stippling indicates that more than 60% of the models agree on the changes in IVT-based events characteristics across CMIP6 models. Inset bar charts in (C) to (F) present IVT-based events characteristics for each risk level. Heatmaps in (C) to (F) show regional relative changes in 30-year windows under ALL+SSP585 during 1921–2100 compared to ALL during 1981–2010 in different climate regions, with "2100" representing the window of 2071–2100. Symbol "▼" indicates climate regions where the |SNR| > 1. Symbol "▼" indicates climate regions where the |SNR| > 1.

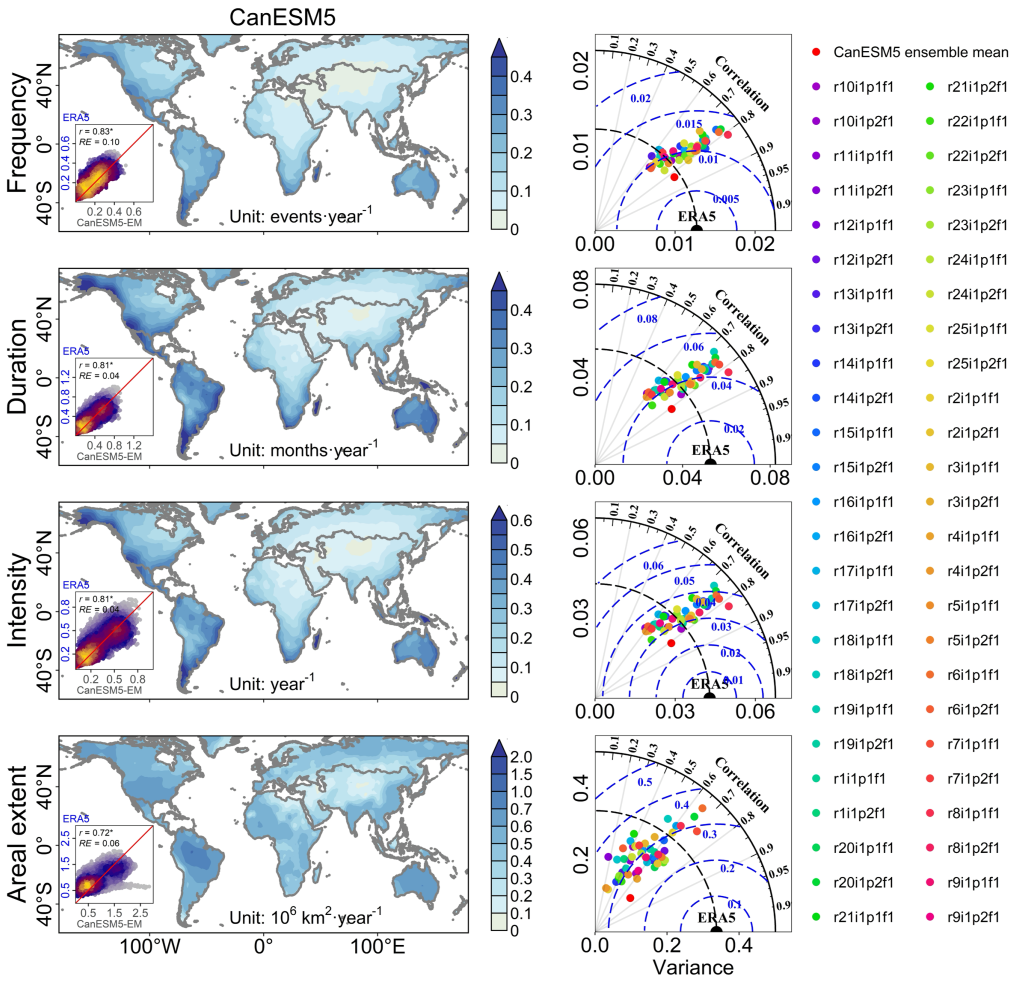

Fig. S10. Climatological characteristics of OWAS during 1961–2020 based on CanESM5. Maps show the frequency, duration, intensity, and areal extent of OWASs based on CanESM5 ensemble mean during 1961–2020. The inset scatterplots represent the OWAS characteristics in each grid cell from CanESM5 ensemble mean and ERA5. The symbol "RE" indicates the relative error. Taylor diagrams show the correlation of OWAS characteristics during 1961–2020 between CanESM5 individual ensemble member and ERA5.

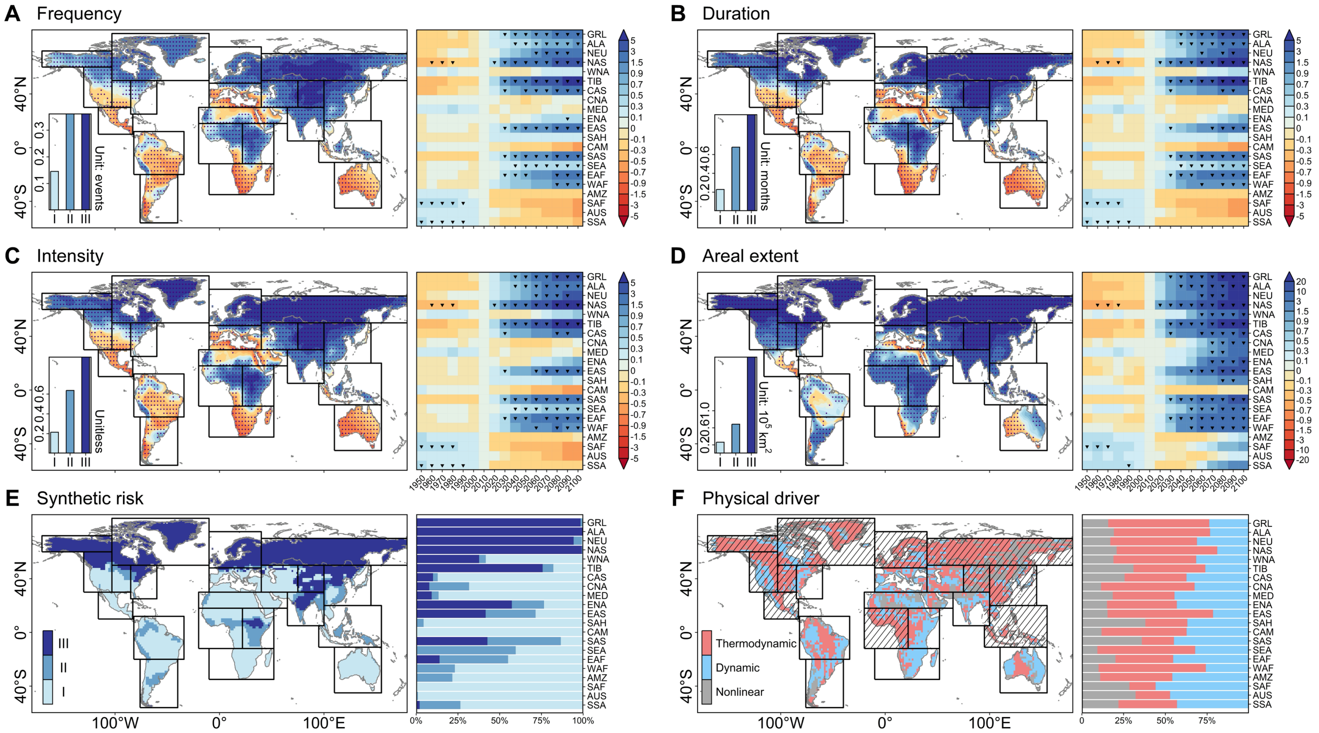

Fig. S11. Projected relative changes in OWAS characteristics under SSP585. (**A**)–(**D**) Maps show projected relative changes in OWAS frequency (A), duration (B), intensity (C), and areal extent (D) between CanESM5 SSP585 during 2071–2100 and ALL during 1981–2010, respectively. Stippling indicates that more than 60% of the ensemble members agree on the changes in OWAS characteristics across CanESM5 ensemble members. Bar charts in maps (A) to (D) present OWAS characteristics for each risk level. Heatmaps accompanying the maps (A) to (D) show regional relative changes in 30-year moving windows under ALL+SSP585 during 1921–2100 compared to ALL during 1981–2010 in different climate regions, with "2100" representing the window of 2071–2100. Symbol "▼" indicates climate regions where the |SNR| > 1. (**E**) Map shows OWAS risk levels globally from CanESM5 SSP585 during 2071–2100. Bar chart represents the proportion of each climate region in different OWAS risk levels. (**F**) Thermodynamic, dynamic, and nonlinear components of normalized differences in moisture flux convergence during OWASs between CanESM5 SSP585 during 2071–2100 and ALL during 1981–2010. Map shows the dominant moisture flux convergence component in each grid cell. Hatchings on the maps show regions where the thermodynamic component dominates. Bar chart shows the contribution of each component in climate region.

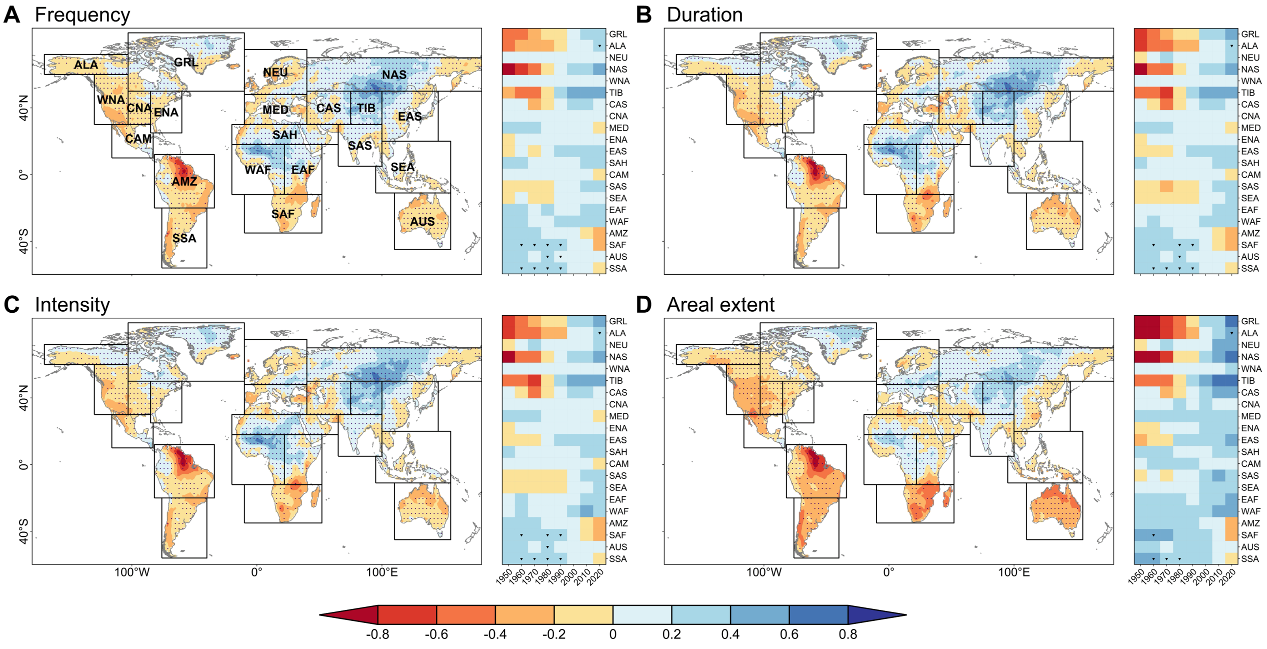

Fig. S12. Human-induced relative changes in OWAS characteristics during 1921–2020. (A)–(D) Maps show the relative changes in frequency (A), duration (B), intensity (C), and areal extent (D) of OWASs based on CanESM5 under GHG effects over the globe during 1981–2010. Stippling indicates that more than 60% of the ensemble members agree on the changes in OWAS characteristics across CanESM5 ensemble members. Black boxes in maps include 21 different Giorgi climate regions. Heatmaps show regional relative changes in OWAS characteristics across 30-year moving windows from 1921 to 2020 under AER compared to under ALL in different climate regions. The label "2020" corresponds to the 1991–2020 window.

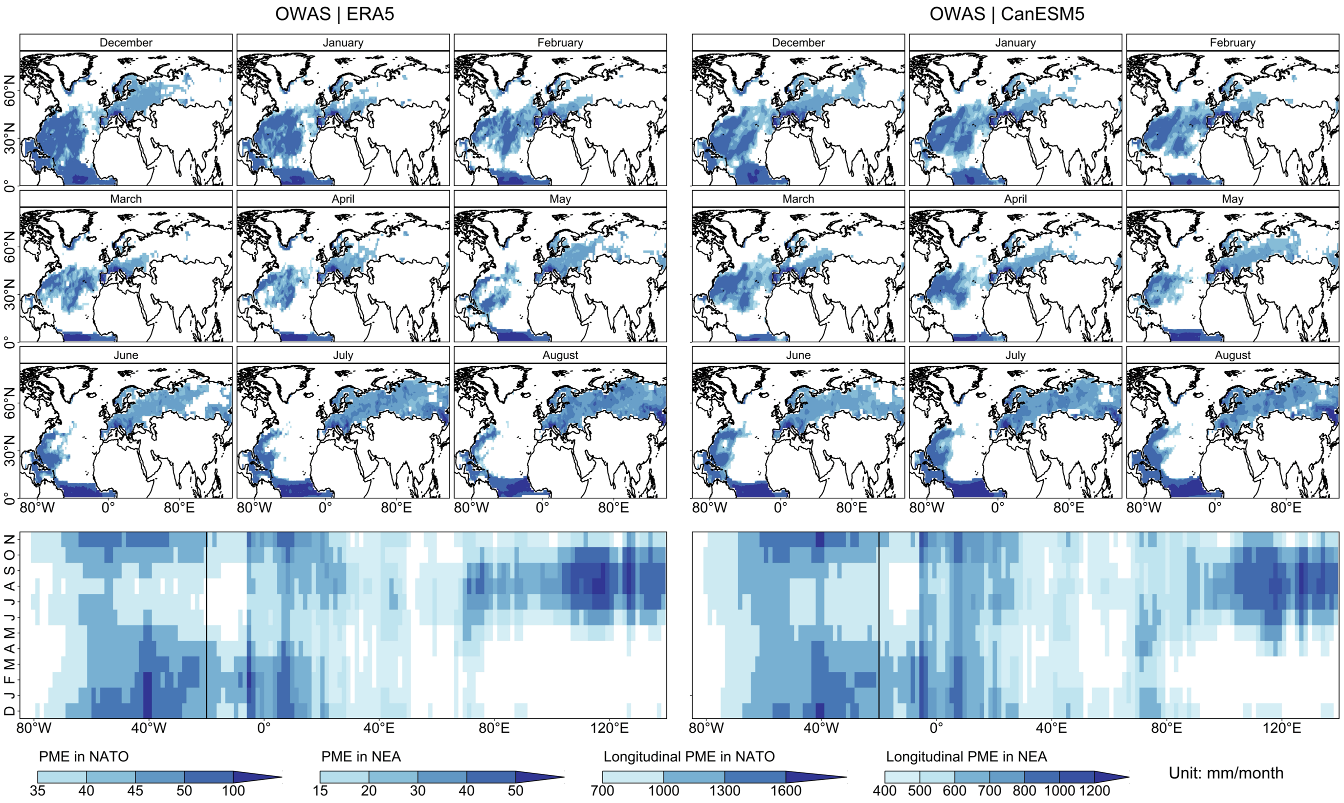

Fig. S13. Landward migration routes of OWASs in NMHL from the northern Atlantic. Maps show water availability anomalies where the standardized water availability index > 0.8 from December to August under ERA5 and CanESM5 ALL during 1961–2020. Heatmap shows longitudinal accumulation of monthly water availability anomalies in the northern Atlantic and northern Eurasia.

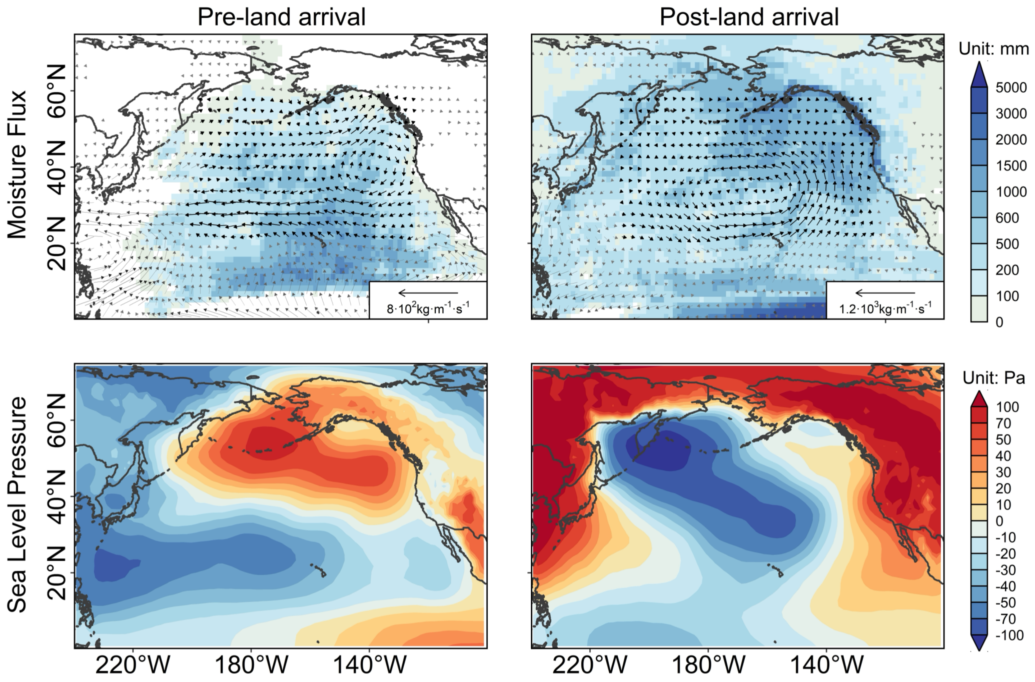

Fig. S14. Atmospheric circulations associated with OWASs in NMHL from northeastern Pacific based on ERA5. Maps show composite anomalies of integrated moisture flux and cumulative water availability anomalies over the northeastern Pacific and western North America between during OWASs (1961–2020) and the climatological mean (1981–2010), as well as composite sea-level pressure anomalies in the northeastern Pacific during the pre-land arrival and post-land arrival period compared to the climatological mean, respectively. The OWAS pre-land arrival period is the period from genesis to arrive at land. The OWAS post-land arrival period is the period from arriving land to extinction.

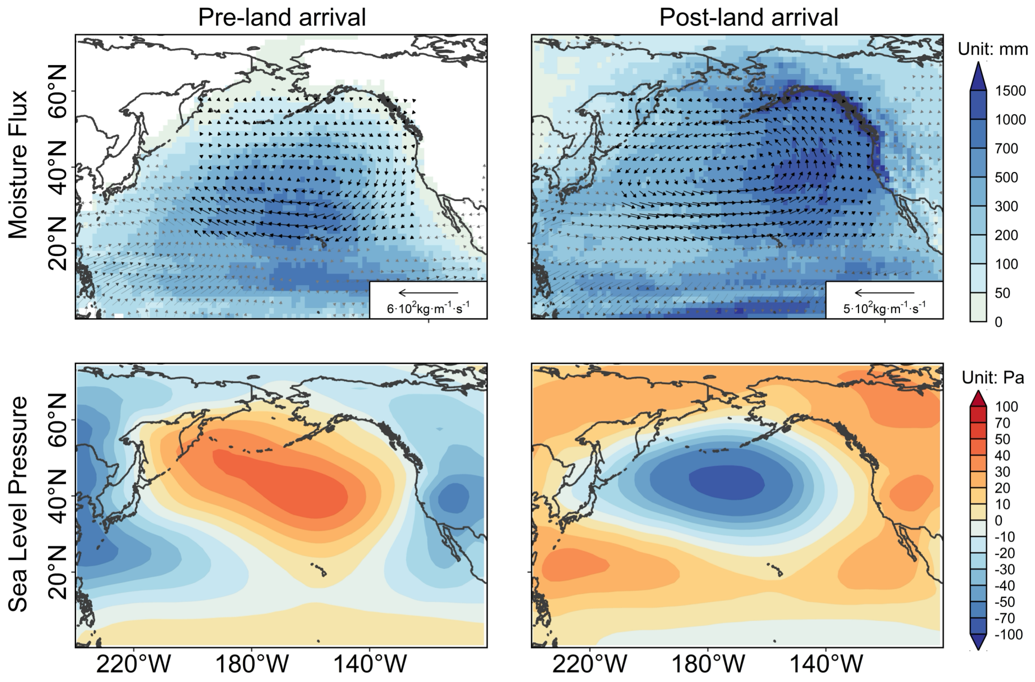

Fig. S15. Atmospheric circulations associated with OWASs in NMHL from northeastern Pacific based on CanESM5. The same as **Fig. S14**, but for CanESM5 ALL.

Supplementary Table

Table S1. CMIP6 simulations and projections used in this study. An asterisk (*) indicates that the corresponding model has the data for a given experiment; a long dash (–) indicates that it does not.

**Model name**	**ALL**	**NAT**	**GHG**	**AER**	**SSP585**
ACCESS-CM2	*	*	*	*	*
ACCESS-ESM1-5	*	*	*	*	*
AWI-CM-1-1-MR	*	–	–	–	*
BCC-CSM2-MR	*	*	*	*	*
CanESM5	*	*	*	*	*
CanESM5-1	*	–	–	–	*
CAS-ESM2-0	*	–	–	–	*
CESM2-WACCM	*	–	–	–	*
CIESM	*	–	–	–	*
CMCC-CM2-SR5	*	–	–	–	*
CMCC-ESM2	*	–	–	–	*
EC-Earth3	*	–	–	–	*
EC-Earth3-CC	*	–	–	–	*
EC-Earth3-Veg	*	–	–	–	*
EC-Earth3-Veg-LR	*	–	–	–	*
FGOALS-f3-L	*	–	–	–	*
FGOALS-g3	*	*	*	*	*
FIO-ESM-2-0	*	–	–	–	*
GFDL-CM4	*	–	–	–	*
GFDL-ESM4	*	*	*	*	*
INM-CM4-8	*	–	–	–	*
INM-CM5-0	*	–	–	–	*
IPSL-CM6A-LR	*	*	*	*	*
KACE-1-0-G	*	–	–	–	*
MIROC6	*	*	*	*	*
MPI-ESM1-2-HR	*	–	–	–	*
MPI-ESM1-2-LR	*	–	–	–	*
MRI-ESM2-0	*	*	*	*	*
NESM3	*	–	–	–	*
NorESM2-LM	*	*	*	*	*
NorESM2-MM	*	–	–	–	*
TaiESM1	*	–	–	–	*
Total counts	32	10	10	10	32

REFERENCES AND NOTES

1. Y. Zhang, C. Li, F. H. S. Chiew, D. A. Post, X. Zhang, N. Ma, J. Tian, D. Kong, L. R. Leung, Q. Yu, J. Shi, C. Liu, Southern hemisphere dominates recent decline in global water availability. *Science* **382**, 579–584 (2023).

2. R. S. Padrón, L. Gudmundsson, B. Decharme, A. Ducharne, D. M. Lawrence, J. Mao, D. Peano, G. Krinner, H. Kim, S. I. Seneviratne, Observed changes in dry-season water availability attributed to human-induced climate change. *Nat. Geosci.* **13**, 477–481 (2020).

3. J. Schewe, J. Heinke, D. Gerten, I. Haddeland, N. W. Arnell, D. B. Clark, R. Dankers, S. Eisner, B. M. Fekete, F. J. Colón-González, S. N. Gosling, H. Kim, X. Liu, Y. Masaki, F. T. Portmann, Y. Satoh, T. Stacke, Q. Tang, Y. Wada, D. Wisser, T. Albrecht, K. Frieler, F. Piontek, L. Warszawski, P. Kabat, Multimodel assessment of water scarcity under climate change. *Proc. Natl. Acad. Sci. U.S.A.* **111**, 3245–3250 (2014).

4. I. Haddeland, J. Heinke, H. Biemans, S. Eisner, M. Flörke, N. Hanasaki, M. Konzmann, F. Ludwig, Y. Masaki, J. Schewe, T. Stacke, Z. D. Tessler, Y. Wada, D. Wisser, Global water resources affected by human interventions and climate change. *Proc. Natl. Acad. Sci. U.S.A.* **111**, 3251–3256 (2014).

5. Y. Guan, X. Gu, L. J. Slater, L. Li, D. Kong, J. Liu, X. Zhang, X. Yan, Tracing anomalies in moisture recycling and transport to two record-breaking droughts over the Mid-to-Lower Reaches of the Yangtze River. *J. Hydrol.* **609**, 127787 (2022).

6. L. Guo, N. P. Klingaman, M.-E. Demory, P. L. Vidale, A. G. Turner, C. C. Stephan, The contributions of local and remote atmospheric moisture fluxes to East Asian precipitation and its variability. *Clim. Dyn.* **51**, 4139–4156 (2018).

7. L. Gimeno, R. Nieto, A. Drumond, R. Castillo, R. Trigo, Influence of the intensification of the major oceanic moisture sources on continental precipitation. *Geophys. Res. Lett.* **40**, 1443–1450 (2013).

8. T. F. Cheng, M. Lu, Global Lagrangian tracking of continental precipitation recycling, footprints, and cascades. *J. Clim.* **36**, 1923–1941 (2023).

9. H. A. Singh, C. M. Bitz, J. Nusbaumer, D. C. Noone, A mathematical framework for analysis of water tracers: Part 1: Development of theory and application to the preindustrial mean state. *J. Adv. Model. Earth Syst.* **8**, 991–1013 (2016).

10. L. Gimeno, M. Vázquez, J. Eiras-Barca, R. Sorí, M. Stojanovic, I. Algarra, R. Nieto, A. M. Ramos, A. M. Durán-Quesada, F. Dominguez, Recent progress on the sources of continental precipitation as revealed by moisture transport analysis. *Earth Sci. Rev.* **201**, 103070 (2020).

11. Q. Zhang, Z. Shen, Y. Pokhrel, D. Farinotti, V. P. Singh, C.-Y. Xu, W. Wu, G. Wang, Oceanic climate changes threaten the sustainability of Asia's water tower. *Nature* **615**, 87–93 (2023).

12. Y. Guan, X. Gu, L. J. Slater, X. Li, J. Li, L. Wang, X. Tang, D. Kong, X. Zhang, Human-induced intensification of terrestrial water cycle in dry regions of the globe. *NPJ Clim. Atmos. Sci.* **7**, 45 (2024).

13. W. Zhang, K. Furtado, P. Wu, T. Zhou, R. Chadwick, C. Marzin, J. Rostron, D. Sexton, Increasing precipitation variability on daily-to-multiyear time scales in a warmer world. *Sci. Adv.* **7**, eabf8021 (2021).

14. W. Zhang, T. Zhou, P. Wu, Anthropogenic amplification of precipitation variability over the past century. *Science* **385**, 427–432 (2024).

15. B. Gu, S. Zhou, B. Yu, K. L. Findell, B. R. Lintner, Multifaceted changes in water availability with a warmer climate. *NPJ Clim. Atmos. Sci.* **8**, 31 (2025).

16. G. Konapala, A. K. Mishra, Y. Wada, M. E. Mann, Climate change will affect global water availability through compounding changes in seasonal precipitation and evaporation. *Nat. Commun.* **11**, 3044 (2020).

17. S. Zhang, L. Zhou, L. Zhang, Y. Yang, Z. Wei, S. Zhou, D. Yang, X. Yang, X. Wu, Y. Zhang, X. Li, Y. Dai, Reconciling disagreement on global river flood changes in a warming climate. *Nat. Clim. Change* **12**, 1160–1167 (2022).

18. S. Zhou, B. Yu, B. R. Lintner, K. L. Findell, Y. Zhang, Projected increase in global runoff dominated by land surface changes. *Nat. Clim. Change* **13**, 442–449 (2023).

19. M. P. Byrne, P. A. O'Gorman, Link between land-ocean warming contrast and surface relative humidities in simulations with coupled climate models. *Geophys. Res. Lett.* **40**, 5223–5227 (2013).

20. J. T. Fasullo, Robust land–ocean contrasts in energy and water cycle feedbacks. *J. Clim.* **23**, 4677–4693 (2010).

21. X. Zhang, J. He, J. Zhang, I. Polyakov, R. Gerdes, J. Inoue, P. Wu, Enhanced poleward moisture transport and amplified northern high-latitude wetting trend. *Nat. Clim. Change* **3**, 47–51 (2013).

22. J. C. Fernández-Alvarez, A. Pérez-Alarcón, J. Eiras-Barca, S. Rahimi, R. Nieto, L. Gimeno, Projected changes in atmospheric moisture transport contributions associated with climate warming in the north atlantic. *Nat. Commun.* **14**, 6476 (2023).

23. R. Fuentes-Franco, D. Docquier, T. Koenigk, K. Zimmermann, F. Giorgi, Winter heavy precipitation events over northern europe modulated by a weaker NAO variability by the end of the 21st century. *NPJ Clim. Atmos. Sci.* **6**, 72 (2023).

24. E. Moreno-Chamarro, L.-P. Caron, P. Ortega, S. Loosveldt Tomas, M. J. Roberts, Can we trust CMIP5/6 future projections of european winter precipitation? *Environ. Res. Lett.* **16**, 054063 (2021).

25. R. Seager, G. A. Vecchi, Greenhouse warming and the 21st century hydroclimate of southwestern north America. *Proc. Natl. Acad. Sci. U.S.A.* **107**, 21277–21282 (2010).

26. Y. Gao, J. Lu, L. R. Leung, Q. Yang, S. Hagos, Y. Qian, Dynamical and thermodynamical modulations on future changes of landfalling atmospheric rivers over western North America. *Geophys. Res. Lett.* **42**, 7179–7186 (2015).

27. Y. Tan, F. Zwiers, S. Yang, C. Li, K. Deng, The role of circulation and its changes in present and future atmospheric rivers over western north America. *J. Clim.* **33**, 1261–1281 (2020).

28. F. Giorgi, R. Francisco, Uncertainties in regional climate change prediction: A regional analysis of ensemble simulations with the HADCM2 coupled AOGCM. *Clim. Dyn.* **16**, 169–182 (2000).

29. V. Eyring, S. Bony, G. A. Meehl, C. A. Senior, B. Stevens, R. J. Stouffer, K. E. Taylor, Overview of the Coupled Model Intercomparison Project Phase 6 (CMIP6) experimental design and organization. *Geosci. Model Dev.* **9**, 1937–1958 (2016).

30. M. Rodell, B. Li, Changing intensity of hydroclimatic extreme events revealed by GRACE and GRACE-FO. *Nat. Water* **1**, 241–248 (2023).

31. B. Fang, M. Lu, Asia faces a growing threat from intraseasonal compound weather whiplash. *Earths Future* **11**, e2022EF003111 (2023).

32. T. Jiang, X. Su, G. Zhang, T. Zhang, H. Wu, Estimating propagation probability from meteorological to ecological droughts using a hybrid machine learning-Copula method. *Hydrol. Earth Syst. Sci.* 10.5194/hess-2022-78 (2022).

33. B. Tellman, J. A. Sullivan, C. Kuhn, A. J. Kettner, C. S. Doyle, G. R. Brakenridge, T. A. Erickson, D. A. Slayback, Satellite imaging reveals increased proportion of population exposed to floods. *Nature* **596**, 80–86 (2021).

34. B. Tellman, J. A. Sullivan, C. Kuhn, A. J. Kettner, C. S. Doyle, G. R. Brakenridge, T. A. Erickson, D. A. Slayback, Global flood database, (2021); http://global-flood-database.cloudtostreet.info/.

35. B. A. Colle, K. Rojowsky, F. Buonaito, New york city storm surges: Climatology and an analysis of the wind and cyclone evolution. *J. Appl. Meteorol. Climatol.* **49**, 85–100 (2010).

36. Z. W. Kundzewicz, S. Kanae, S. I. Seneviratne, J. Handmer, N. Nicholls, P. Peduzzi, R. Mechler, L. M. Bouwer, N. Arnell, K. Mach, R. Muir-Wood, G. R. Brakenridge, W. Kron, G. Benito, Y. Honda, K. Takahashi, B. Sherstyukov, Flood risk and climate change: Global and regional perspectives. *Hydrol. Sci. J.* **59**, 1–28 (2014).

37. J. Liu, X. Feng, X. Gu, J. Zhang, L. J. Slater, D. Kong, Detection and attribution of human influence on the global diurnal temperature range decline. *Geophys. Res. Lett.* **49**, e2021GL097155 (2022).

38. A. J. Cannon, S. R. Sobie, T. Q. Murdock, Bias correction of GCM precipitation by quantile mapping: How well do methods preserve changes in quantiles and extremes? *J. Clim.* **28**, 6938–6959 (2015).

39. X. Yuan, Y. Jiao, D. Yang, H. Lei, Reconciling the attribution of changes in streamflow extremes from a hydroclimate perspective. *Water Resour. Res.* **54**, 3886–3895 (2018).

40. I. M. Held, B. J. Soden, Robust responses of the hydrological cycle to global warming. *J. Clim.* **19**, 5686–5699 (2006).

41. J. He, B. J. Soden, A re-examination of the projected subtropical precipitation decline. *Nat. Clim. Change* **7**, 53–57 (2017).

42. Y. Yao, D. Luo, Relationship between zonal position of the north atlantic oscillation and euro-atlantic blocking events and its possible effect on the weather over europe. *Sci. China Earth Sci.* **57**, 2628–2636 (2014).

43. F. Lienert, F. J. Doblas-Reyes, Prediction of interannual north atlantic sea surface temperature and its remote influence over land. *Clim. Dyn.* **48**, 3099–3114 (2017).

44. H.-M. Kim, M. A. Alexander, ENSO's modulation of water vapor transport over the pacific–north American region. *J. Clim.* **28**, 3846–3856 (2015).

45. H.-M. Kim, Y. Zhou, M. A. Alexander, Changes in atmospheric rivers and moisture transport over the northeast pacific and western north America in response to ENSO diversity. *Clim. Dyn.* **52**, 7375–7388 (2019).

46. K. Marvel, B. I. Cook, C. J. W. Bonfils, P. J. Durack, J. E. Smerdon, A. P. Williams, Twentieth-century hydroclimate changes consistent with human influence. *Nature* **569**, 59–65 (2019).

47. C. Chou, J. D. Neelin, C.-A. Chen, J.-Y. Tu, Evaluating the "rich-get-richer" mechanism in tropical precipitation change under global warming. *J. Clim.* **22**, 1982–2005 (2009).

48. R. Seager, N. Naik, G. A. Vecchi, Thermodynamic and dynamic mechanisms for large-scale changes in the hydrological cycle in response to global warming. *J. Clim.* **23**, 4651–4668 (2010).

49. R. Seager, D. Neelin, I. Simpson, H. Liu, N. Henderson, T. Shaw, Y. Kushnir, M. Ting, B. Cook, Dynamical and thermodynamical causes of large-scale changes in the hydrological cycle over north America in response to global warming. *J. Clim.* **27**, 7921–7948 (2014).

50. M. Newman, G. N. Kiladis, K. M. Weickmann, F. M. Ralph, P. D. Sardeshmukh, Relative contributions of synoptic and low-frequency eddies to time-mean atmospheric moisture transport, including the role of atmospheric rivers. *J. Clim.* **25**, 7341–7361 (2012).

51. M. P. Byrne, P. A. O'Gorman, The response of precipitation minus evapotranspiration to climate warming: Why the "wet-get-wetter, dry-get-drier" scaling does not hold over land. *J. Clim.* **28**, 8078–8092 (2015).

52. L. Gimeno, J. Eiras-Barca, A. M. Durán-Quesada, F. Dominguez, R. Van Der Ent, H. Sodemann, R. Sánchez-Murillo, R. Nieto, J. W. Kirchner, The residence time of water vapour in the atmosphere. *Nat. Rev. Earth Environ.* **2**, 558–569 (2021).

53. L. Gimeno, A. Drumond, R. Nieto, R. M. Trigo, A. Stohl, On the origin of continental precipitation. *Geophys. Res. Lett.* **37**, 2010GL043712 (2010).

54. L. Bakels, D. Tatsii, A. Tipka, R. Thompson, M. Dütsch, M. Blaschek, P. Seibert, K. Baier, S. Bucci, M. Cassiani, S. Eckhardt, C. Groot Zwaaftink, S. Henne, P. Kaufmann, V. Lechner, C.

Maurer, M. D. Mulder, I. Pisso, A. Plach, R. Subramanian, M. Vojta, A. Stohl, FLEXPART version 11: Improved accuracy, efficiency, and flexibility. *Geosci. Model Dev.* **17**, 7595–7627 (2024).

55. Z. Shen, Q. Zhang, V. P. Singh, Y. Pokhrel, J. Li, C.-Y. Xu, W. Wu, Drying in the low-latitude Atlantic Ocean contributed to terrestrial water storage depletion across Eurasia. *Nat. Commun.* **13**, 1849 (2022).

56. G. Wang, Q. Zhang, Y. Pokhrel, D. Farinotti, J. Wang, V. P. Singh, C.-Y. Xu, Exogenous moisture deficit fuels drought risks across China. *npj Clim. Atmos. Sci.* **6**, 217 (2023).

57. H. Paltan, D. Waliser, W. H. Lim, B. Guan, D. Yamazaki, R. Pant, S. Dadson, Global floods and water availability driven by atmospheric rivers. *Geophys. Res. Lett.* **44**, 10387–10395 (2017).

58. Y. Lai, J. Li, X. Gu, Y. D. Chen, D. Kong, T. Y. Gan, M. Liu, Q. Li, G. Wu, Greater flood risks in response to slowdown of tropical cyclones over the coast of China. *Proc. Natl. Acad. Sci. U.S.A.* **117**, 14751–14755 (2020).

59. A. Khouakhi, G. Villarini, G. A. Vecchi, Contribution of tropical cyclones to rainfall at the global scale. *J. Clim.* **30**, 359–372 (2017).

60. A. Webb, T. Shimura, N. Mori, Global tropical cyclone track detection and analysis of the d4PDF mega-ensemble projection. *J. Jpn. Soc. Civ. Eng. Ser. B2 Coast. Eng.* **75**, 1207–1212 (2019).

61. S. J. Camargo, A. A. Wing, Increased tropical cyclone risk to coasts. *Science* **371**, 458–459 (2021).

62. Y. Li, Y. Tang, S. Wang, R. Toumi, X. Song, Q. Wang, Recent increases in tropical cyclone rapid intensification events in global offshore regions. *Nat. Commun.* **14**, 5167 (2023).

63. R. Mo, EDARA: An ERA5-based dataset for atmospheric river analysis. *Sci Data* **11**, 900 (2024).

64. A. E. Payne, M.-E. Demory, L. R. Leung, A. M. Ramos, C. A. Shields, J. J. Rutz, N. Siler, G. Villarini, A. Hall, F. M. Ralph, Responses and impacts of atmospheric rivers to climate change. *Nat. Rev. Earth Environ.* **1**, 143–157 (2020).

65. J. T. Pasquier, S. Pfahl, C. M. Grams, Modulation of atmospheric river occurrence and associated precipitation extremes in the north atlantic region by european weather regimes. *Geophys. Res. Lett.* **46**, 1014–1023 (2019).

66. I. Algarra, R. Nieto, A. M. Ramos, J. Eiras-Barca, R. M. Trigo, L. Gimeno, Significant increase of global anomalous moisture uptake feeding landfalling Atmospheric Rivers. *Nat. Commun.* **11**, 5082 (2020).

67. X. Chen, T. Zhou, P. Wu, Z. Guo, M. Wang, Emergent constraints on future projections of the western north pacific subtropical high. *Nat. Commun.* **11**, 2802 (2020).

68. K. Yang, W. Cai, G. Huang, K. Hu, B. Ng, G. Wang, Increased variability of the western Pacific subtropical high under greenhouse warming. *Proc. Natl. Acad. Sci. U.S.A.* **119**, e2120335119 (2022).

69. N. C. Swart, J. N. S. Cole, V. V. Kharin, M. Lazare, J. F. Scinocca, N. P. Gillett, J. Anstey, V. Arora, J. R. Christian, S. Hanna, Y. Jiao, W. G. Lee, F. Majaess, O. A. Saenko, C. Seiler, C. Seinen, A. Shao, M. Sigmond, L. Solheim, K. Von Salzen, D. Yang, B. Winter, The Canadian Earth System Model version 5 (CanESM5.0.3). *Geosci. Model Dev.* **12**, 4823–4873 (2019).

70. J. Liu, S. Feng, X. Gu, Y. Zhang, H. E. Beck, J. Zhang, S. Yan, Global changes in floods and their drivers. *J. Hydrol.* **614**, 128553 (2022).

71. J. Yin, P. Gentine, S. Zhou, S. C. Sullivan, R. Wang, Y. Zhang, S. Guo, Large increase in global storm runoff extremes driven by climate and anthropogenic changes. *Nat. Commun.* **9**, 4389 (2018).

72. J. T. Reager, J. S. Famiglietti, Global terrestrial water storage capacity and flood potential using GRACE. *Geophys. Res. Lett.* **36**, 2009GL040826 (2009).

73. J. Yin, S. Guo, P. Gentine, S. C. Sullivan, L. Gu, S. He, J. Chen, P. Liu, Does the hook structure constrain future flood intensification under anthropogenic climate warming? *Water Resour. Res.* **57**, e2020WR028491 (2021).

74. L. Slater, G. Villarini, S. Archfield, D. Faulkner, R. Lamb, A. Khouakhi, J. Yin, Global changes in 20-year, 50-year, and 100-year river floods. *Geophys. Res. Lett.* **48**, e2020GL091824 (2021).

75. W. Liu, T. Yang, F. Sun, H. Wang, Y. Feng, M. Du, Observation-constrained projection of global flood magnitudes with anthropogenic warming. *Water Resour. Res.* **57**, e2020WR028830 (2021).

76. B. Asadieh, N. Y. Krakauer, Global change in streamflow extremes under climate change over the 21st century. *Hydrol. Earth Syst. Sci.* **21**, 5863–5874 (2017).

77. H. Hersbach, B. Bell, P. Berrisford, S. Hirahara, A. Horányi, J. Muñoz-Sabater, J. Nicolas, C. Peubey, R. Radu, D. Schepers, A. Simmons, C. Soci, S. Abdalla, X. Abellan, G. Balsamo, P. Bechtold, G. Biavati, J. Bidlot, M. Bonavita, G. Chiara, P. Dahlgren, D. Dee, M. Diamantakis, R. Dragani, J. Flemming, R. Forbes, M. Fuentes, A. Geer, L. Haimberger, S. Healy, R. J. Hogan, E. Hólm, M. Janisková, S. Keeley, P. Laloyaux, P. Lopez, C. Lupu, G. Radnoti, P. Rosnay, I. Rozum, F. Vamborg, S. Villaume, J. Thépaut, The ERA5 global reanalysis. *Q. J. R. Meteorol. Soc.* **146**, 1999–2049 (2020).

78. Copernicus Climate Change Service, ERA5 monthly averaged data on single levels from 1940 to present, Copernicus Climate Change Service (C3S) Climate Data Store (CDS) (2019); https://doi.org/10.24381/CDS.F17050D7.

79. Copernicus Climate Change Service, ERA5 monthly averaged data on pressure levels from 1940 to present, Copernicus Climate Change Service (C3S) Climate Data Store (CDS) (2019); https://doi.org/10.24381/CDS.6860A573.

80. R. Gelaro, W. McCarty, M. J. Suárez, R. Todling, A. Molod, L. Takacs, C. A. Randles, A. Darmenov, M. G. Bosilovich, R. Reichle, K. Wargan, L. Coy, R. Cullather, C. Draper, S. Akella, V. Buchard, A. Conaty, A. M. Da Silva, W. Gu, G.-K. Kim, R. Koster, R. Lucchesi, D. Merkova, J. E. Nielsen, G. Partyka, S. Pawson, W. Putman, M. Rienecker, S. D. Schubert, M. Sienkiewicz,

B. Zhao, The Modern-Era Retrospective Analysis for Research and Applications, Version 2 (MERRA-2). *J. Clim.* **30**, 5419–5454 (2017).

81. S. Kobayashi, Y. Ota, Y. Harada, A. Ebita, M. Moriya, H. Onoda, K. Onogi, H. Kamahori, C. Kobayashi, H. Endo, K. Miyaoka, K. Takahashi, The JRA-55 reanalysis: General specifications and basic characteristics. *J. Meteorol. Soc. Jpn.* **93**, 5–48 (2015).

82. J. Yin, L. J. Slater, A. Khouakhi, L. Yu, P. Liu, F. Li, Y. Pokhrel, P. Gentine, GTWS-MLrec: Global terrestrial water storage reconstruction by machine learning from 1940 to present. *Earth Syst. Sci. Data* **15**, 5597–5615 (2023).

83. R. Mo, An ERA5-based dataset for atmospheric river analysis (EDARA): Multi-decade numerical and graphical catalogues, Federated Research Data Repository/dépôt fédéré de données de recherche (2024); https://doi.org/10.20383/103.0935.

84. B. Guan, D. E. Waliser, Tracking atmospheric rivers globally: Spatial distributions and temporal evolution of life cycle characteristics. *J. Geophys. Res. Atmos.* **124**, 12523–12552 (2019).

85. N. P. Gillett, H. Shiogama, B. Funke, G. Hegerl, R. Knutti, K. Matthes, B. D. Santer, D. Stone, C. Tebaldi, The Detection and Attribution Model Intercomparison Project (DAMIP v1.0) contribution to CMIP6. *Geosci. Model Dev.* **9**, 3685–3697 (2016).

86. M. G. Donat, A. L. Lowry, L. V. Alexander, P. A. O’Gorman, N. Maher, More extreme precipitation in the world’s dry and wet regions. *Nat. Clim. Change* **6**, 508–513 (2016).

87. S. Zhou, A. P. Williams, B. R. Lintner, K. L. Findell, T. F. Keenan, Y. Zhang, P. Gentine, Diminishing seasonality of subtropical water availability in a warmer world dominated by soil moisture-atmosphere feedbacks. *Nat. Commun.* **13**, 5756 (2022).

88. N. Maher, S. Milinski, R. Ludwig, Large ensemble climate model simulations: Introduction, overview, and future prospects for utilising multiple types of large ensemble. *Earth Syst. Dynam.* **12**, 401–418 (2021).

89. F. Piontek, T. Geiger, ISIMIP2b secondary population input data, version 1.0, ISIMIP Repository (2024); https://doi.org/10.48364/ISIMIP.432399.

90. K. Klein Goldewijk, A. Beusen, P. Janssen, Long-term dynamic modeling of global population and built-up area in a spatially explicit way: HYDE 3.1. *Holocene* **20**, 565–573 (2010).

91. D. P. Van Vuuren, K. Riahi, K. Calvin, R. Dellink, J. Emmerling, S. Fujimori, S. Kc, E. Kriegler, B. O'Neill, The shared socio-economic pathways: Trajectories for human development and global environmental change. *Glob. Environ. Change* **42**, 148–152 (2017).

92. Y. Guan, X. Gu, L. J. Slater, J. Yin, J. Li, S. H. Gebrechorkos, X. Zhang, D. Kong, X. Yan, Increase in ocean-onto-land droughts and their drivers under anthropogenic climate change. *npj Clim. Atmos. Sci.* **6**, 195 (2023).

93. L. Samaniego, R. Kumar, M. Zink, Implications of parameter uncertainty on soil moisture drought analysis in Germany. *J. Hydrometeorol.* **14**, 47–68 (2013).

94. J. Sheffield, A simulated soil moisture based drought analysis for the United States. *J. Geophys. Res.* **109**, D24108 (2004).

95. L. Samaniego, S. Thober, R. Kumar, N. Wanders, O. Rakovec, M. Pan, M. Zink, J. Sheffield, E. F. Wood, A. Marx, Anthropogenic warming exacerbates European soil moisture droughts. *Nat. Clim. Change* **8**, 421–426 (2018).

96. J. E. Herrera-Estrada, N. S. Diffenbaugh, Landfalling droughts: Global tracking of moisture deficits from the oceans onto land. *Water Resour. Res.* **56**, e2019WR026877 (2020).

97. K. M. Andreadis, E. A. Clark, A. W. Wood, A. F. Hamlet, D. P. Lettenmaier, Twentieth-century drought in the conterminous United States. *J. Hydrometeorol.* **6**, 985–1001 (2005).

98. J. E. Herrera-Estrada, J. Sheffield, Uncertainties in future projections of summer droughts and heat waves over the contiguous United States. *J. Clim.* **30**, 6225–6246 (2017).

99. H. Vernieuwe, B. De Baets, N. E. C. Verhoest, A mathematical morphology approach for a qualitative exploration of drought events in space and time. *Int. J. Climatol.* **40**, 530–543 (2020).

100. J. Sheffield, K. M. Andreadis, E. F. Wood, D. P. Lettenmaier, Global and continental drought in the second half of the twentieth century: Severity–area–duration analysis and temporal variability of large-scale events. *J. Clim.* **22**, 1962–1981 (2009).

101. J. E. Herrera-Estrada, Y. Satoh, J. Sheffield, Spatiotemporal dynamics of global drought. *Geophys. Res. Lett.* **44**, 2254–2263 (2017).

102. X. Yuan, Y. Wang, P. Ji, P. Wu, J. Sheffield, J. A. Otkin, A global transition to flash droughts under climate change. *Science* **380**, 187–191 (2023).

103. X. Gu, Z. Jiang, Y. Guan, M. Luo, J. Li, L. Wang, X. Zhang, D. Kong, L. Wang, Frequent land-ocean transboundary migration of tropical heatwaves under climate change. *Nat. Commun.* **16**, 3400 (2025).

104. T. Zhou, L. Ren, W. Zhang, Anthropogenic influence on extreme Meiyu rainfall in 2020 and its future risk. *Sci. China Earth Sci.* **64**, 1633–1644 (2021).

105. X. Tan, X. Wu, Z. Huang, J. Fu, X. Tan, S. Deng, Y. Liu, T. Y. Gan, B. Liu, Increasing global precipitation whiplash due to anthropogenic greenhouse gas emissions. *Nat. Commun.* **14**, 2796 (2023).

106. S. Zhou, A. P. Williams, B. R. Lintner, A. M. Berg, Y. Zhang, T. F. Keenan, B. I. Cook, S. Hagemann, S. I. Seneviratne, P. Gentine, Soil moisture–atmosphere feedbacks mitigate declining water availability in drylands. *Nat. Clim. Change* **11**, 38–44 (2021).

107. S. H. Baek, J. M. Lora, Counterbalancing influences of aerosols and greenhouse gases on atmospheric rivers. *Nat. Clim. Change* **11**, 958–965 (2021).

108. M. K. Roxy, S. Ghosh, A. Pathak, R. Athulya, M. Mujumdar, R. Murtugudde, P. Terray, M. Rajeevan, A threefold rise in widespread extreme rain events over central India. *Nat. Commun.* **8**, 708 (2017).

109. M. Hauser, A. Spring, J. Busecke, M. van Driel, R. Lorenz, readthedocs-assistant, regionmask/regionmask: Version 0.12.1, version v0.12.1, Zenodo (2024); https://doi.org/10.5281/ZENODO.10849860.

110. M.-T. Anan, M. Alawad, B. Alsaeed, MB: The use of marginal distributions in conditional forecasting, (2022); https://doi.org/10.32614/CRAN.package.MB.

111. The scikit-learn developers, scikit-learn, version 1.7.0, Zenodo (2025); https://doi.org/10.5281/ZENODO.15606905.

112. B. Brentan, G. Meirelles, E. Luvizotto, J. Izquierdo, Hybrid SOM+*k*-means clustering to improve planning, operation and management in water distribution systems. *Environ. Model. Softw.* **106**, 77–88 (2018).

113. P. Zeng, F. Sun, Y. Liu, Y. Wang, G. Li, Y. Che, Mapping future droughts under global warming across China: A combined multi-timescale meteorological drought index and SOM-*K*means approach. *Weather Clim. Extrem.* **31**, 100304 (2021).

114. Y. Markonis, R. Kumar, M. Hanel, O. Rakovec, P. Máca, A. AghaKouchak, The rise of compound warm-season droughts in Europe. *Sci. Adv.* **7**, eabb9668 (2021).

115. L. J. Harrington, P. B. Gibson, S. M. Dean, D. Mitchell, S. M. Rosier, D. J. Frame, Investigating event-specific drought attribution using self-organizing maps. *J. Geophys. Res. Atmos.* **121**, 12766–12780 (2016).

116. L. A. Parsons, S. Coats, Ocean-atmosphere trajectories of extended drought in Southwestern North America. *J. Geophys. Res. Atmos.* **124**, 8953–8971 (2019).

117. T. Kohonen, Essentials of the self-organizing map. *Neural Netw.* **37**, 52–65 (2013).

118. J. A. Francis, N. Skific, Z. Zobel, Weather whiplash events in europe and north atlantic assessed as continental-scale atmospheric regime shifts. *npj Clim. Atmos. Sci.* **6**, 216 (2023).

119. N. Godin, S. Huguet, R. Gaertner, Integration of the kohonen's self-organising map and *k*-means algorithm for the segmentation of the AE data collected during tensile tests on cross-ply composites. *NDT Int.* **38**, 299–309 (2005).

120. D. L. Davies, D. W. Bouldin, A cluster separation measure. *IEEE Trans. Pattern Anal. Mach. Intell.* **PAMI-1**, 224–227 (1979).

121. J. Olsson, K. Berggren, M. Olofsson, M. Viklander, Applying climate model precipitation scenarios for urban hydrological assessment: A case study in Kalmar City, Sweden. *Atmos. Res.* **92**, 364–375 (2009).

122. P. Willems, M. Vrac, Statistical precipitation downscaling for small-scale hydrological impact investigations of climate change. *J. Hydrol.* **402**, 193–205 (2011).

123. G. Bürger, T. Q. Murdock, A. T. Werner, S. R. Sobie, A. J. Cannon, Downscaling extremes—An intercomparison of multiple statistical methods for present climate. *J. Clim.* **25**, 4366–4388 (2012).

124. E. Hawkins, R. Sutton, The potential to narrow uncertainty in regional climate predictions. *Bull. Am. Meteorol. Soc.* **90**, 1095–1108 (2009).
